# The Effect of Lipid-Lowering Therapy on Coronary Artery Plaque in East Asia Population

**DOI:** 10.1016/j.jacasi.2025.05.016

**Published:** 2025-07-15

**Authors:** Tomohiro Fujisaki, Yuichiro Shirahama, Feng Sheng, Ken Ikeda, Nobuhiro Osada, Satoru Tanaka, Yang Zhang, Kenichi Tsujita

**Affiliations:** a Department of Cardiovascular Medicine, Graduate School of Medical Sciences, Kumamoto University, Kumamoto, Japan; b Department of Cardiovascular Medicine, National Cerebral and Cardiovascular Center, Suita, Osaka, Japan; c Amgen K.K., Tokyo, Japan; d Systematic Review Solutions Ltd, Shanghai, China

**Keywords:** coronary atherosclerotic plaque, East Asian population, lipid-lowering therapy, meta-analysis, progression, stability

## Abstract

**Background:**

Lipid-lowering therapy (LLT) is important to control atherosclerosis and prevent atherosclerotic cardiovascular disease (ASCVD) events.

**Objectives:**

This study aimed to summarize the effects of LLT on coronary atherosclerotic plaque stability and progression in East Asian individuals.

**Methods:**

A systematic literature search was performed from respective inception dates to November 21, 2023. Studies carried out on East Asian participants who received LLT were included. Meta-analysis and meta-regression analysis were used to investigate the effects of LLT on plaque regression indicators and plaque stabilization indicators (PROSPERO registration number CRD42024504184).

**Results:**

Forty-eight studies with a total of 4,147 patients were included in the final analysis. In East Asian patients, low-density lipoprotein cholesterol (LDL-C) levels ≤130 mg/dL resulted in a pooled percent atheroma volume decrease of −1.09% (95% CI: −1.38% to −0.79%; *I*^2^ = 91%), and the lowest levels of LDL-C (≤55 mg/dL) were associated with the greatest decrease (−1.56%, 95% CI: −2.20% to −0.92%; *I*^2^ = 0%) of percent atheroma volume when compared with levels in the range of 55 to 70, 70 to 100, and 100 to 130 mg/dL. LLT resulted in a pooled fibrous cap thickness gain of 66.90 μm (95% CI: 50.06-83.75 μm) at the end of the follow-up. There was a trend that decreasing follow-up LDL-C levels were associated with larger increases of fibrous cap thickness, especially when LCL-C was <70 mg/dL.

**Conclusions:**

LLT is beneficial for East Asian patients with established ASCVD or with higher risks of ASCVD. Intensive LLT with a lower target LDL-C, especially to <55 mg/dL, would be beneficial for atherosclerosis treatment.

Cardiovascular diseases (CVDs) remain the leading causes of death worldwide.^1^^,^^2^ Atherosclerotic CVD (ASCVD) is the main component of CVD. The development and progression of atherosclerotic plaque (AP) is the core pathophysiological mechanism of ASCVD. High-risk coronary atherosclerotic plaque (CAP), which is characterized by a large lipid pool covered with a thin fibrous cap, is the main cause of sudden cardiac death, acute coronary syndrome, and ischemic stroke.^3^^,^^4^

In recent years, high-resolution intraluminal imaging techniques, including intravascular ultrasound (IVUS) and intravascular optical coherence tomography (OCT), have enabled the direct and precise assessment of CAP.^5^^,^^6^ Indicators of CAP including percent atheroma volume (PAV), total atheroma volume (TAV), and plaque volume (PV) have been widely used to assess plaque burden.^7^ Compared with OCT, IVUS has a deeper radial investigation depth that enables it to measure the whole wall of the target artery segment.^5^^,^^6^ Meanwhile, OCT has a resolution of 10 μm which enables it to easily identify vulnerable plaques and their composition.^8^^,^^9^ Near-infrared spectroscopy (NIRS) is designed for the detection of the lipid core in CAP, allowing it to provide an assessment of the characteristics of plaque, including composition and inflammation within the plaque.^6^ Indicators evaluated from OCT and NIRS, such as lipid core burden index (LCBI), fibrous cap thickness (FCT), lipid arc, and macrophages, have been widely employed to evaluate the stability of CAP.

Elevated levels of low-density lipoprotein cholesterol (LDL-C) are a major risk factor for CAP and a pivotal therapeutic target in cardiovascular event prevention.^10^ Reducing LDL-C is associated with the decrease of PAV and increase of the FCT, which indicates the regression and stabilization of CAP.^11^, ^12^, ^13^, ^14^ A significant decrease in serum level of LDL-C is associated with improved prognosis in patients with ASCVD.^15^, ^16^, ^17^ With the accumulation of evidence supporting the benefits of LDL-C lipid-lowering therapy (LLT) for participants with established ASCVD or higher risks of ASCVD, drugs including statins, ezetimibe, and proprotein convertase subtilisin kexin 9 inhibitors (PCSK9i) have been recommended for primary and secondary prevention of ASCVD.^18^ A meta-regression analysis showed that a 1% decline in mean PAV induced by lipid treatment was associated with approximately a 20% reduction in the odds of major adverse cardiovascular events.^19^^,^^20^ Recent developments in LLTs have shown that non-statin therapies, such as PCSK9i and ezetimibe, may be effective in reducing LDL-C levels and improving clinical outcomes in patients with atherosclerosis.^21^^,^^22^ To delay the progression of CAP and lower the occurrence of adverse events, the latest guidelines recommend that patients with a very high risk of ASCVD should aim to lower their LDL-C levels to <55 mg/dL, or even <40 mg/dL in the case of recurrence within 2 years of an acute coronary syndrome (ACS) event.^23^^,^^24^

Similar to other parts of the world, East Asia has a heavy burden of ASCVD.^25^ However, the evidence supporting the current lipid treatment thresholds recommended in guidelines mostly comes from studies in Western countries. The biological and genetic profiles of Asian ethnicities are significantly different from those of Caucasians, and the common aggregation of the “Asian” population might also mask the differences in the lipid profiles, statin responses, and lipid targets.^26^ East Asian populations have generally been prescribed lower doses due to their superior statin responsiveness and lower LDL-C levels when compared with Western populations.^27^, ^28^, ^29^ In a recent study on noncalcified plaque among Caucasian and East Asian patients with suspected coronary artery disease (CAD), East Asian patients’ plaque comprised less high-risk (fibrofatty and necrotic core) plaque and more low-risk (fibrous) plaque.^30^ This highlights the importance of summarizing the results of related studies on the response to LLT in East Asian individuals. It also emphasizes the need to provide evidence for the development of tailored treatment strategies to improve cardiovascular outcomes in this population. The primary aim of the present study, therefore, was to provide this summary and evidence.

## Methods

The systematic review and meta-analysis in this study were performed following the Preferred Reporting Items for Systematic Reviews and Meta-Analyses (PRISMA) guidelines.^31^ A comprehensive protocol was prospectively submitted and registered in PROSPERO before commencing the study and analysis (registration number CRD42024504184).

### Search strategy

A literature search was performed in PubMed, EMBASE, Cochrane Library, and Web of Science from respective inception dates to November 21, 2023. The search strategy mainly covered the following criteria: 1) East Asian population (including individuals from Mainland China, Hongkong, Macao, Taiwan, South Korea, Japan, Mongolia, and North Korea); 2) interventions of interest, including statins, ezetimibe, PCSK9i, and a combination of these drugs; and 3) study design, including randomized controlled trials (RCTs) and observational studies. The search strategy was initially developed for PubMed and then adapted appropriately for other databases. There were no restrictions on publication language. After retrieving the articles, the reference lists of selected studies and previous systematic reviews were manually reviewed. Detailed search strategies are listed in Supplemental File 1.

### Study selection

Two reviewers screened all retrieved references and selected studies that met the inclusion criteria by following 2 steps: 1) screening title and abstracts; and then 2) reading the full-text articles of all potentially relevant citations where available. Discrepancies were resolved by discussion or with assistance from a third party if necessary. A PRISMA flow diagram was constructed to show the full study-selection process.

#### Study eligibility

The inclusion criteria were as follows: 1) population, interventions, and study designs were described previously in the “search strategy” section; 2) comparators: not limited; 3) outcomes: plaque regression examined by IVUS or OCT, with indicators including TAV (including PV) and PAV; and 4) plaque stability examined by OCT or NIRS, with indicators including LCBI, FCT, and LDL-C level. The exclusion criteria were as follows: 1) non-East Asian populations; 2) patients not receiving LLT; 3) studies that reported only plaque-related outcomes or only LDL-C; and 4) abstract-only search results

### Data extraction

Two reviewers independently extracted data using a standardized data extraction form. The following information was collected when available: demographic characteristics (e.g., age, sex, country), study design, description of the intervention, sample size, outcomes, measures, and results. The primary outcomes of this study included PAV and FCT. The secondary outcomes included TAV (including PV) and LCBI. Discrepancies were resolved by discussion or, if necessary, referral to a third reviewer.

### Assessment of methodological quality

Two reviewers independently assessed the risk of bias in the included studies. We used the Cochrane risk-of-bias tool to assess the risk of bias of the RCTs^32^ and the Newcastle-Ottawa Scale for Non-Randomized Studies of Interventions.^33^ We evaluated every risk-of-bias domain, based on the standard criteria (Before-After [Pre-Post]) outlined by the National Institutes of Health, to assess the before-and-after studies.^34^ Disagreements were resolved by discussion, with assistance from a third party if necessary.

### Data synthesis

#### Meta-analysis

R software (version 4.4.3) was used to perform the statistical analysis. A random-effects model was used, taking into consideration the differences in diseases or interventions between the included studies. We used the mean and standard deviations (SDs) to describe the continuous outcomes. If the mean or SDs were not reported and not available, data that were available such as the median values and CIs were used; the mean and SD values were then re-calculated from the information recorded in the study. A random-effects model was used to calculate the combined mean and 95% CI. The results were presented as a pooled analysis in forest plots. The Cochran’s Q test and *I*^2^ statistics were used to examine heterogeneity across studies, where *P* values <0.10 and *I*^2^ >50% were considered significant for heterogeneity.^35^ If *I*^2^ >50%, we explored the source of the heterogeneity using a sensitivity analysis.

Considering that the purpose of this study was to identify the LDL-C targets and LDL-C change values for plaque regression and plaque stability, subgroup analyses of LDL-C levels were performed. Stratified analyses according to the levels of LDL-C at follow-up (≤55, 55-70, 70-100, 100-130, and >130 mg/dL), the follow-up LDL-C changes from the baseline (≤1 mmol/L and >1 mmol/L), and drug administration regimens on the primary outcomes of PAV and FCT were performed.

#### Meta-regression

Given the potential influence of confounding factors on the study’s results, we investigated the relationship between the dependent variable and the covariate through meta-regression analysis. We used the Restricted Maximum Likelihood method for the random-effects model in meta-regression. We hypothesized that the included studies may have shown differences according to the LDL-C, background disease, age, sex, drugs and drug dosages, treatment duration, smoking, diabetes, hypertension, and blood lipids of the patients. To evaluate the possible impact of these factors on the meta-analysis results, a regression model with the PAV or FCT as the dependent variable (y) and the previously mentioned covariate as the independent variable (x) was established. In addition, a regression model with the TAV or LCBI as the dependent variable (y) and the LDL-C as the independent variable (x) was established. All statistical analyses were performed using R 4.1.1 with meta and Metafor commands.

#### Publication bias

Publication bias was quantitatively examined by Egger’s test and funnel plots when the number of included studies reached 10 or more.^36^

## Results

### Characteristics of included studies

**Figure 1:**
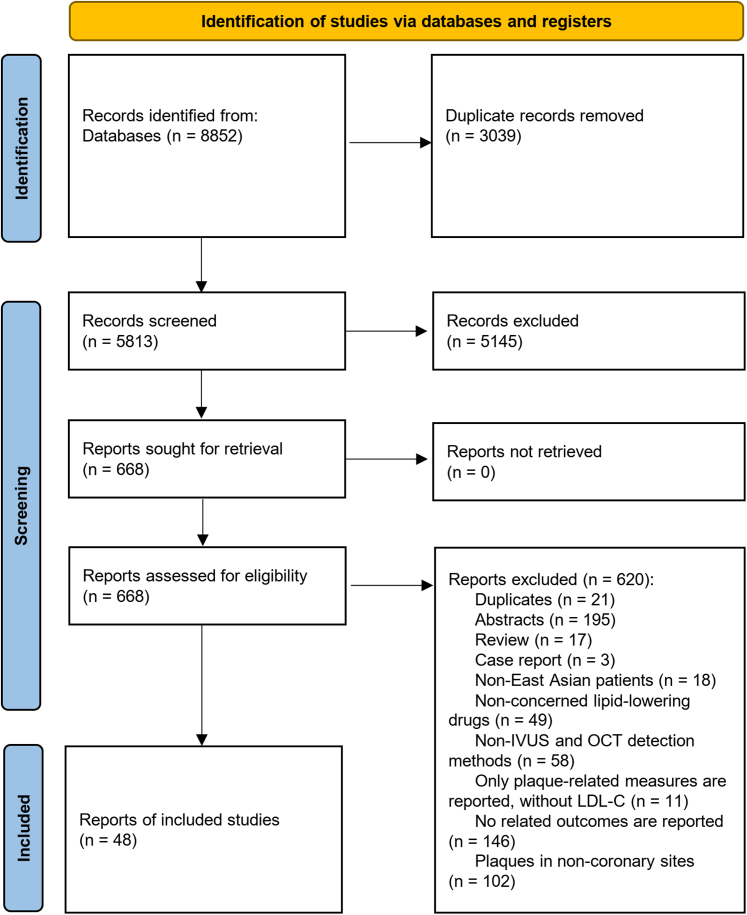
Preferred Reporting Items for Systematic Reviews and Meta-Analyses Flowchart of Included Studies A total of 48 studies from 48 articles were included in the final analysis. IVUS = intravascular ultrasound; LDL-C = low-density lipoprotein cholesterol; OCT = optical coherence tomography.

A total of 48 studies from 48 articles (see Supplemental File 1) were included in the final analysis (Figure 1), of which 26 were from Japan, 12 were from China, and 10 were from South Korea. These studies included 29 RCTs, 10 cohort studies, 3 clinical control trials, 3 single-arm trials, 2 post hoc analyses, and 1 case-control prospective study. Sample sizes ranged from 20 to 271, with a total of 4,147 patients involved across the studies. Forty-five studies reported the mean of age, with the mean age ranging from 52.1 to 77 years. Combined LLTs were used in 14 studies, of which 9 used ezetimibe combined with statins, and 5 used PCSK9i combined with statins. Treatment duration was reported in 47 studies. IVUS was used in 38 studies, OCT was used in 14 studies, and NIRS was used in 1 study. Baseline LDL-C level was reported in 47 studies, and follow-up change of LDL-C was reported in 41 studies. Baseline FCT was reported in 9 studies. Detailed information included in the studies is listed in Supplemental File 2.

### Plaque regression

#### PAV-related analysis

##### Effect of follow-up LDL-C levels or of LDL-C levels on PAV

Overall, LLTs were associated with a pooled PAV decrease of −1.09% (95% CI: −1.38% to −0.79%; *I*^2^ = 91%) at the end of the follow-up (Supplemental Figure 1). The lowest level of follow-up LDL-C (≤55 mg/dL) was associated with the greatest decrease (−1.56%; 95% CI: −2.20% to −0.92%; *I*^2^ = 0%) of PAV when compared with level ranges 55-70 (−1.00%; 95% CI: −1.33% to −0.67%; *I*^2^ = 81%), 70-100 (−1.03%; 95% CI: −1.53% to −0.53%; *I*^2^ = 94%), and 100-130 mg/dL (0.95%; 95% CI: −1.25% to 3.15%; *I*^2^ = 91%), although there were no significant differences between the groups (*P* = 0.128) (Supplemental Figure 2). However, meta-regression analysis did not show significant association between the follow-up absolute value of LDL-C level and the change in PAV (Supplemental Figure 3, Supplemental Table 1).

The pooled decrease (−1.30%; 95% CI: −1.73% to −0.87%; *I*^2^ = 93%) of PAV in patients with a reduction of LDL-C level >38.67 mmol/L (1 mmol/L = 38.67 mg/dL) was significantly higher (*P* = 0.027) than that (−0.65%; 95% CI: −1.03% to −0.27%; *I*^2^ = 85%) in patients with a reduction of LDL-C level ≤38.67 mg/dL (Supplemental Figure 4). Furthermore,meta-regression analysis showed that a 1-mg/dL increase in LDL-C level change was associated with a 0.03% decrease in PAV (Supplemental Figure 5, Supplemental Table 1).

A high degree of heterogeneity was observed in these analyses, and the source of heterogeneity was explored unfolding that the study by Hiro et al^37^ was a source of significant heterogeneity (Supplemental Figure 6). Due to its remarkable heterogeneity among the included studies, we excluded the Hiro et al^37^ study in PAV-related analyses.

**Figure 2:**
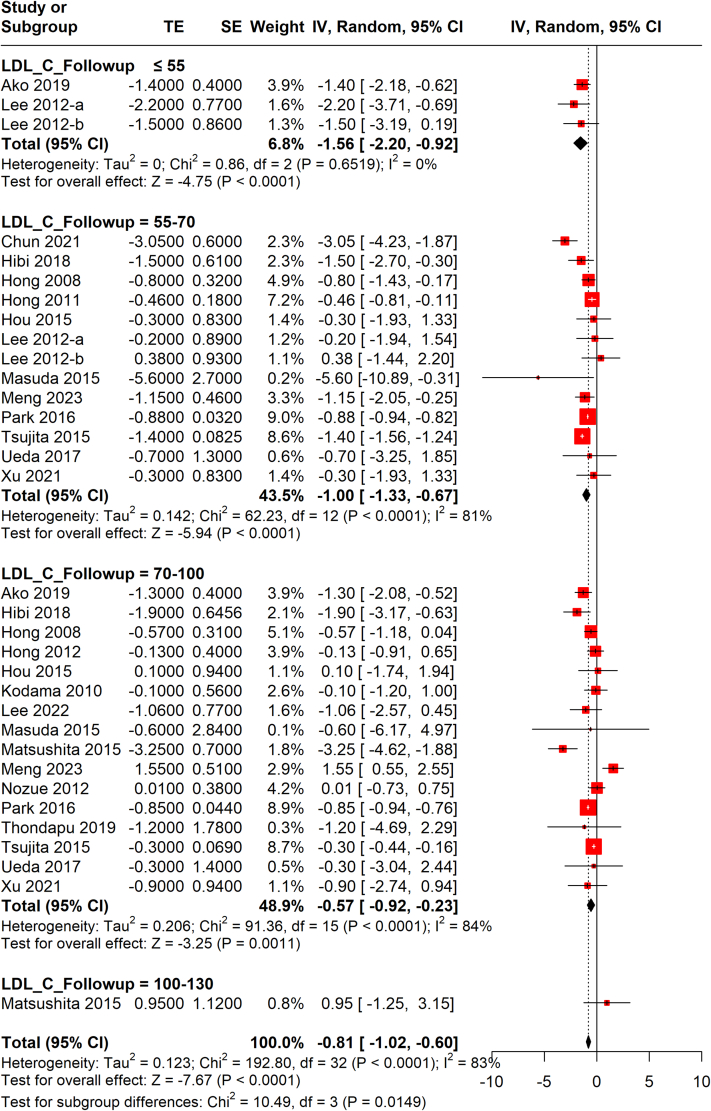
Random-Effects Meta-analysis of PAV Changes by Follow-Up LDL-C Levels Meta-analysis revealed that the lowest level of follow-up LDL-C (≤55 mg/dL) was associated with the greatest decrease of PAV when compared with level ranges of 55 to 70, 70 to 100, and 100 to 130 mg/dL (Hiro et al^37^ study excluded). IV = inverse variance; PAV = percent atheroma volume; SE = standard error; TE = treatment effect; other abbreviation as in Figure 1.

**Figure 3:**
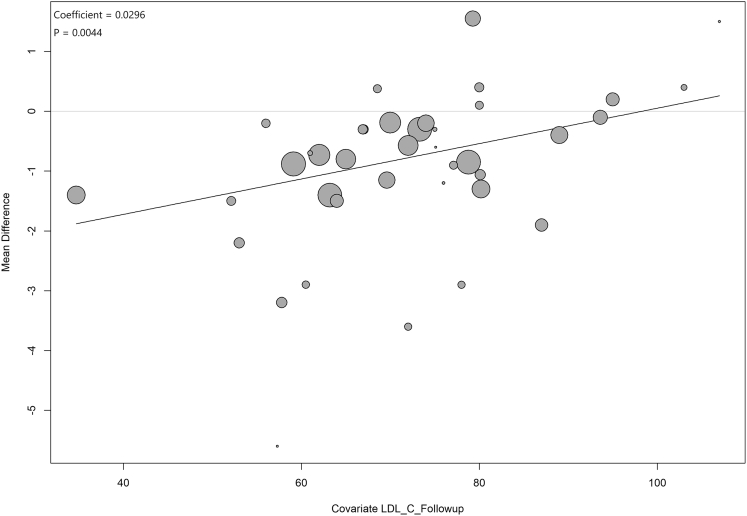
Meta-Regression of PAV Changes on Follow-Up LDL-C Levels Meta-regression analysis also showed that a 1-mg/dL reduction of absolute LDL-C level at follow-up resulted in a 0.03% decrease in PAV (Hiro et al^37^ study excluded). Abbreviations as in Figures 1 and 2.

After excluding the Hiro et al^37^ study from the current analyses, a linear trend between follow-up LDL-C levels and PAV was observed, and subgroup differences stratified by follow-up LDL-C levels became significant (*P* = 0.015) (Figure 2). Meta-regression analysis also showed that a 1-mg/dL reduction of absolute LDL-C level at follow-up resulted in a 0.03% decrease in PAV (Figure 3, Supplemental Table 2).

The pooled decrease (−0.91%; 95% CI: −1.18% to −0.63%; *I*^2^ = 75%) of PAV in patients with a reduction of LDL-C level >38.67 mg/dL was higher than that (−0.65%; 95% CI: −1.03% to −0.27%; *I*^2^ = 85%) in patients with a reduction of LDL-C level ≤38.67 mg/dL, which was concordant with the principal analysis (Supplemental Figure 7). Meta-regression analysis showed that a 1-mg/dL increase in LDL-C level change was associated with a 0.02% decrease in PAV (Supplemental Figure 8, Supplemental Table 2).

##### Effect of different regimens on PAV

**Figure 4:**
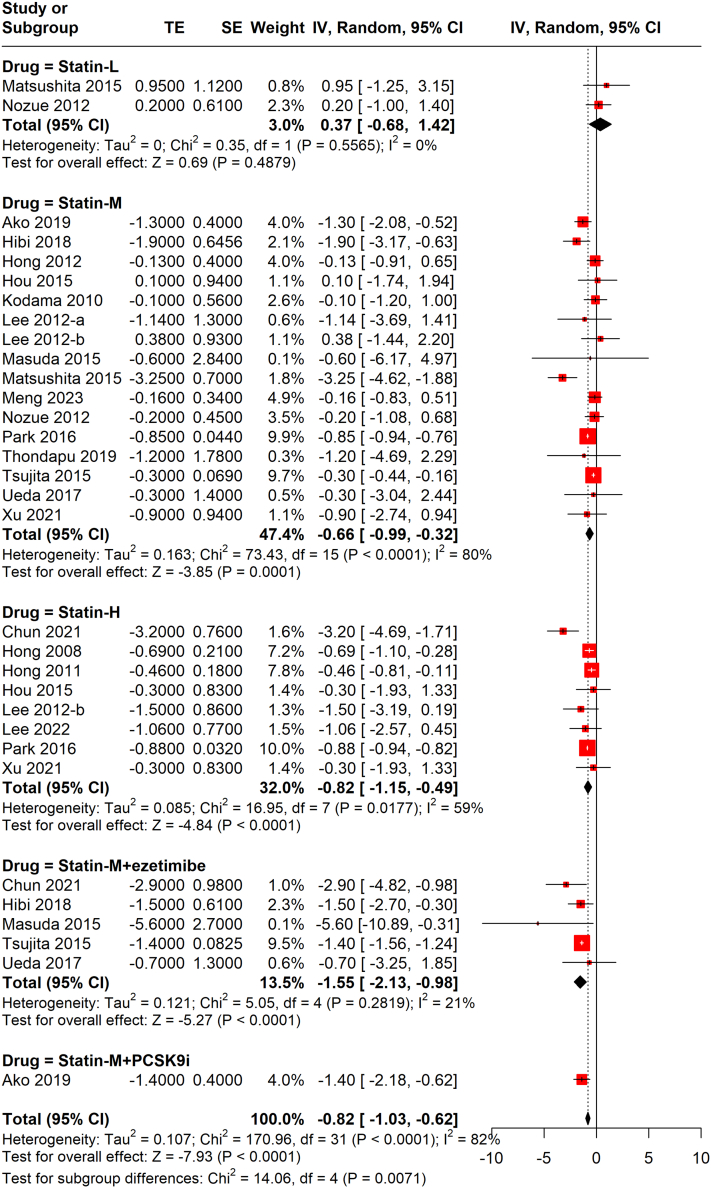
Random-Effects Meta-Analysis of PAV Changes by Regimen Combination lipid-lowering therapy such as moderate-intensity statin combined with ezetimibe or PCSK9i were associated with the highest decreases of PAV, with ezetimibe in 5 studies and PCSK9i in 1 study, followed by statin of high intensity, moderate intensity, and low intensity. PCSK9i = proprotein convertase subtilisin kexin 9 inhibitors; other abbreviations as in Figures 1 and 2.

Combination LLTs such as moderate-intensity statin combined with ezetimibe (−1.55%; 95% CI: −2.13% to −0.98%; *I*^2^ = 21%) or PCSK9i (−1.40%; 95% CI: −2.18% to −0.62%) were associated with the highest decreases of PAV, with ezetimibe in 5 studies and PCSK9i in 1 study, followed by statin of high intensity (−0.82%; 95% CI: −1.15% to −0.49%; *I*^2^ = 59%), moderate intensity (−0.66%; 95% CI: −0.99% to −0.32%; *I*^2^ = 80%), and low intensity (0.37%; 95% CI: −0.68% to 1.42%) (Figure 4). A significant difference between subgroups was observed (*P* = 0.007).

Further, subgroup analysis according to different treatment duration revealed that 8 months and 10 months of low-intensity statin therapy both had no significant influence on PAV (Supplemental Figure 9), whereas longer duration of moderate-intensity (10 months and 12 months, but not 6 to 9 months, Supplemental Figure 10) and high-intensity (11 months and 12 months, but not 6 months, Supplemental Figure 11) statin therapy significantly decreased PAV, and moderate-intensity statin + ezetimibe therapy decreased PAV significantly in each duration of 6, 9, 10, and 12 months (Supplemental Figure 12).

##### Effect of other factors on PAV

We further investigated whether there were some other factors associated with PAV change after LLT. The results showed that in patients treated with LLT, none of the major clinical characteristics, including type of disease (ACS or CAD), sex, age, smoking status, hypertension, diabetes, HbA1C, high-density lipoprotein cholesterol (HDL-C), baseline LDL-C, and treatment duration of LLT, were statistically associated with PAV change (Supplemental Table 3). None of the major study characteristics, including publication years, country, and study design were statistically associated with PAV change (Supplemental Table 3).

#### TAV-related analysis

We also analyzed the change of TAV and associated factors at the end of the follow-up, in which TAV reached a pooled reduction of 3.78 mm^3^ (95% CI: −5.37 to −2.18; *I*^2^ = 91%) (Supplemental Figure 13). Meta-regression analysis revealed that a 1-mg/dL reduction of absolute LDL-C level at follow-up resulted in a 0.15 mm^3^ decrease of TAV and a 1-mg/dL of increase in LDL-C level change resulted in a 0.19 mm^3^ reduction of TAV (Supplemental Figures 14 and 15).

### Plaque stability

#### FCT-related analysis

##### Effect of LDL-C levels on FCT

**Figure 5:**
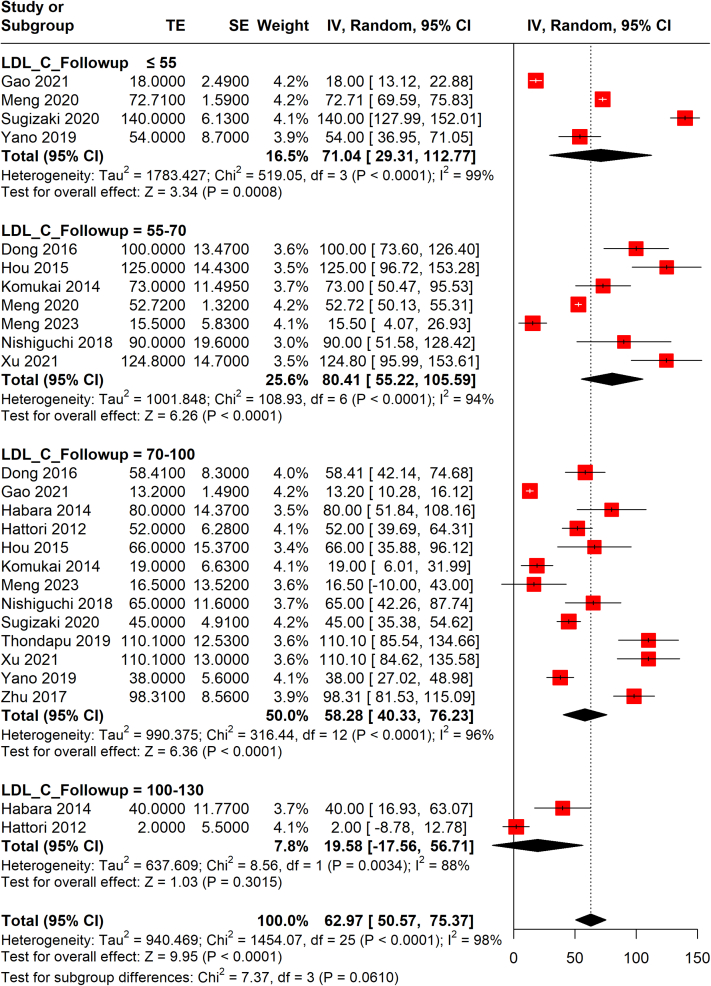
Random-Effects Meta-Analysis of FCT Changes by Follow-Up LDL-C Levels Lower follow-up LDL-C levels were associated with increased FCTs, especially when LCL-C was <70 mg/dL, but not with statistical significance. FCT = fibrous cap thickness; other abbreviations as in Figures 1 and 2.

**Figure 6:**
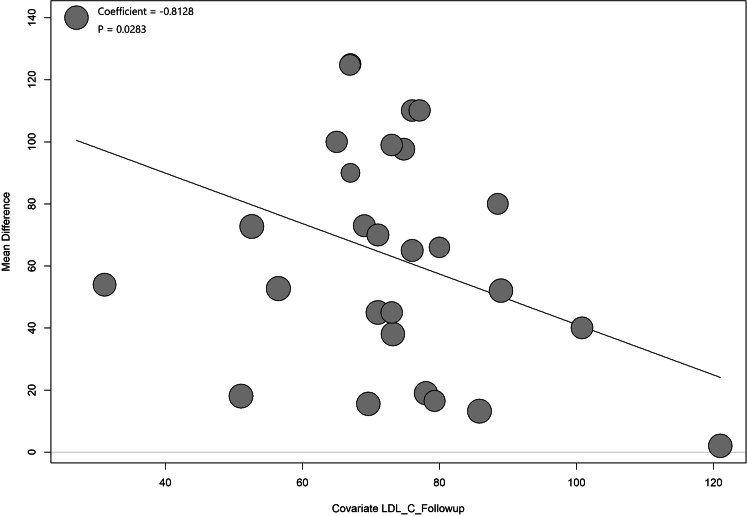
Meta-Regression of Fibrous Cap Thickness Changes on Follow-Up LDL-C Levels Meta-regression analysis revealed that a 1-mg/dL decrease in follow-up LDL-C levels resulted in a 0.81-μm increase of FCT. Abbreviation as in Figure 1.

LLTs were associated with a pooled FCT increase of 66.90 μm (95% CI: 50.06-83.75μm; *I*^2^ = 99%) at the end of the follow-up (Supplemental Figure 16). The results showed that lower follow-up LDL-C levels were associated with increased FCTs, especially when LCL-C was <70 mg/dL, but not with statistical significance (subgroup difference *P* = 0.061) (Figure 5). However, the meta-regression analysis revealed that 1-mg/dL decrease in follow-up LDL-C levels resulted in a 0.81-μm increase of FCT (*P* = 0.028) (Figure 6, Supplemental Table 4). Although a larger LDL-C change (>1 mmol/L) showed a tendency for thicker FCT than a smaller LDL-C change (<1 mmol/L), there was no significant statistical difference between the 2 groups (subgroup difference *P* = 0.214) (Supplemental Figure 17) and meta-regression analysis also showed no significant correlation between LDL-C level change and FCT change (Supplemental Figure 18).

##### Effect of different regimens on FCT

**Figure 7:**
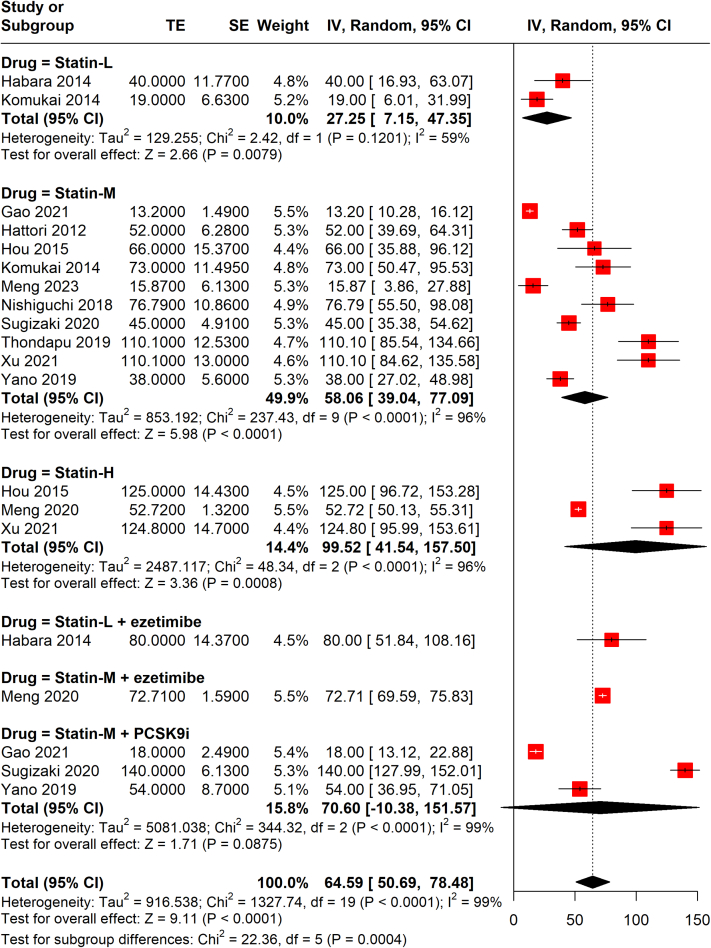
Random-Effects Meta-Analysis of FCT Changes by Regimen High-intensity statin alone was associated with the highest FCT changes at the end of the follow-up, followed by statin combined with ezetimibe and statin combined with PCSK9i. Low-intensity statin was associated with the lowest change of FCT. Abbreviations as in Figures 1, 2, 4, and 5.

A comparison of the impact of different statin-based LLTs on FCT changes found that high-intensity statin alone was associated with the highest FCT changes at the end of the follow-up, followed by statin combined with ezetimibe and statin combined with PCSK9i. Low-intensity statin was associated with the lowest change of FCT (Figure 7). A significant difference between subgroups was observed (*P* = 0.0004).

In a subgroup analysis of changes in FCT according to treatment regimen and duration, moderate-intensity statin therapy at longer durations was associated with higher changes of FCT. No significant differences of FCT changes were observed for the other regimens and durations (Supplemental Figures 19 to 21).

##### Effect of other factors on FCT

Further meta-regression analysis found that CAD, sex, age, follow-up levels, and level changes of HDL-C, and baseline FCT were all significantly associated with FCT change at the end of the follow-up (Supplemental Table 5). Based on this finding, we conducted a subgroup analysis according to the previously mentioned factors and found that patients with CAD (compared with patients with ACS), with an age range of 50 to 60 years (compared with 60 to 70 years), and a baseline FCT <65 μm (compared with more than 65 μm) all had higher FCT changes at the end of the follow-up (Supplemental Table 6).

Further, analyses investigating the effects of LDL-C on FCT changes in different subgroups found that lower follow-up LDL-C levels were associated with higher FCT changes in patients with CAD. Of note, LDL-C changes >1 mmol/L were only associated with higher FCT changes in ACS patients and not CAD patients (Supplemental Table 7).

The sex subgroups analysis revealed that, in studies in which 40% to 70% were male, patients with follow-up LDL-C levels of 55 to 70 mg/dL had the highest FCT change when compared with patients with LDL-C ≤55 mg/dL or >70 mg/dL. Meanwhile, in studies in which more than 70% of patients were male, LDL-C changes >1 mmol/L were associated with higher FCT changes (Supplemental Table 8).

According to age subgroups, among patients of 60 to 70 years, LDL-C change >1 mmol/L was associated with higher FCT change (Supplemental Table 9).

In the change of HDL-C subgroups, in patients with HDL-C changes that decreased or stayed unchanged from the baseline, lower follow-up LDL-C levels were associated with higher FCT changes (Supplemental Table 10).

For patients with baseline FCT >65 μm or <65 μm, no significant difference was found in FCT changes in subgroup analysis according to LDL-C follow-up levels or change values (Supplemental Table 11).

In short, a follow-up LDL-C level of 55 to 70 mg/dL was associated with the highest changes in FCT across most subgroups. However, no significant FCT changes were observed between different LDL-C absolute changes across most subgroups, except in patients with ACS, groups in which more than 70% of patients were male, and patients of 60 to 70 years.

None of the major study characteristics, including publication years, country, and study design were statistically associated with FCT change (Supplemental Table 5).

#### LCBI-related analysis

At the end of the follow-up, the pooled mean difference of LCBI was −42.39 (−127.17 to 42.40; *I*^2^ = 92%) (*P* > 0.05, Supplemental Figure 22). However, meta-regression analysis was not feasible because only 2 studies were available regarding LCBI.

### Sensitivity analysis and publication bias

A sensitivity analysis was conducted by performing additional meta-analyses after deleting individual studies one by one. As mentioned previously, the heterogeneity in PAV originated from the study by Hiro et al,^37^ whereas no sources of heterogeneity were identified in the analyses related to FCT and TAV (Supplemental Figures 23 and 24). No significant publication bias was found in this analysis (Supplemental Figures 25 to 27).

## Discussion

This study analyzed the effect of LLT on PAV, TAV, FCT, and LCBI in the East Asian population based on meta-analysis and meta-regression analysis of the included studies (Central Illustration). We found that the lower the follow-up level of LDL-C, the greater the decrease of PAV. However, no significantly different decrease of PAV was found between changes of LDL-C level ≤1 mmol/L and >1 mmol/L from the baseline. Further analysis revealed that high-intensity LLT was associated with a greater decrease of PAV when compared with low-intensity LLT. We also found that LLT was associated with the decrease of TAV, and both follow-up LDL-C level and the change of LDL-C level had a significant relationship with the decrease of TAV. Further, follow-up LDL-C is significantly associated with FCT. There was no significant relationship between FCT and different changes in LDL-C levels.

**Central Illustration:**
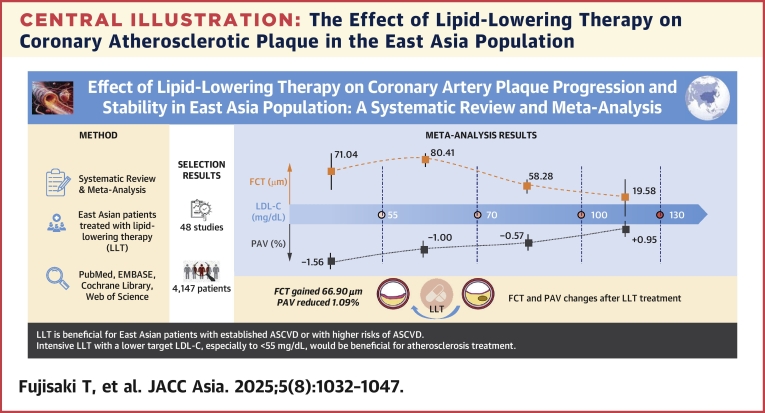
The Effect of Lipid-Lowering Therapy on Coronary Atherosclerotic Plaque in the East Asia Population This meta-analysis of 48 studies illustrates the benefits of lipid-lowering therapy for East Asian patients with established ASCVD or at high ASCVD risk. ASCVD = atherosclerotic cardiovascular disease; FCT = fibrous cap thickness; LDL-C = low-density lipoprotein cholesterol; LLT = lipid-lowering therapy; PAV = percent atheroma volume.

PAV and FCT are the most used indicators for CAP in clinical studies. PAV provides a general assessment of plaque burden, and FCT is often used to evaluate the stability of CAP.^14^^,^^38^, ^39^, ^40^ Many previous studies have demonstrated that the benefits of LLTs brought to patients with ASCVD or to individuals at high risk of ASCVD was because of the impacts of LLTs on PAV and FCT. In the present study, we found that when compared with the change of LDL-C, the absolute level of LDL-C at follow-up seemed to be more associated with the impacts of LLTs on CAP. We found that in the East Asian population, follow-up LDL-C ≤55 mg/dL was associated with the highest percentage change in PAV, and LDL-C ≤70 mg/dL was associated with the greatest increase of FCT. These results are consistent with the target LDL-C values set in previous guidelines from Western countries and suggest that LDL-C ≤55 mg/dL also should be recommended as the target level for East Asian patients with ASCVD.

PAV is an important indicator of plaque burden^41^, ^42^, ^43^ and is significantly associated with major adverse cardiovascular events.^44^ However, in some previous literature, authors have argued that high-intensity statin or LLT might not be necessary for patients from East Asia.^45^^,^^46^ In practice, many clinicians did not prescribe high-intensity statin or LLT for patients with established ASCVD or higher risks of ASCVD.^47^ On the other hand, some patients were not willing to receive or were prone to discontinue high-intensity statin or LLT because of financial problems or the fear of side effects.^48^, ^49^, ^50^, ^51^, ^52^, ^53^ The present study provides evidence to demonstrate that East Asian patients could benefit from intensive LLT, including high-intensity statins or statin therapy combined with PCSK9i or ezetimibe.

In the present study, we did not identify any other risk factors associated with PAV besides LDL-C and LLT. However, meta-regression analysis showed that CAD, sex, age, HDL-C change value, and baseline FCT were all significantly associated with the changes in FCT. In subgroup analyses of the changes in FCT according to the previously mentioned factors, we found that patients with CAD (compared with ACS), in the age range of 50 to 60 years (compared with 60 to 70 years), or with a baseline FCT <65 μm (compared with >65 μm), had a larger increase of FCT. This finding suggests that other factors besides LDL-C and LLT also affect plaque stabilization. Interestingly, in the present study, the change of FCT in patients with follow-up LDL-C levels ≤55 mg/dL was lower than in patients with follow-up LDL-C levels of 55 to 70 mg/dL (71.04 μm vs 80.41 μm), although without statistical significance, which seemed contradictory to expectations. It is possible that patients with follow-up LDL-C levels ≤55 mg/dL had higher baseline FCTs, because our study also found that patients with lower baseline FCTs had greater changes of FCT. Further, it seemed that high-intensity statin alone was associated with a greater change of FCT when compared with statin-based combination LLTs, although the CIs overlapped widely, which would need to be further investigated. This result might be explained by the small number of studies reporting the impacts of high-intensity statin, low-intensity statin, ezetimibe, or PCSK9i on FCT, especially due to the later approval of PCSK9i and a large disparity in treatment effects between studies in clinical use. Notably, in a recent study by Uehara et al,^54^ the results showed that the changes in minimal FCT from baseline over a 9-month period were significantly greater in the PCSK9i group, even after PCSK9i discontinuation. Similar results were also observed in another study.^42^

Although the significant difference in the impact of treatment duration on the change of FCT was only observed in patients treated with moderate statin, we still observed a trend in which increasing durations of LLT resulted in higher changes of FCT across different LLT regimens. This trend supported long-term LLT for patients with established or at high risk of ASCVD.^55^ In all of the included FCT-related studies, the longest treatment duration was approximately 12 months. This suggests that although patients from East Asia might have some racial differences from Western populations,^56^ it was beneficial for them to receive 12 months of intensive treatment for plaque stabilization.

This study has a number of important strengths. First, this study was a large-scale meta-analysis of 4,147 East Asian patients and was specifically designed to investigate the effects of LLT on CAP stability and progression using comprehensive subgroup analyses. Our study focused on East Asian patients, distinguishing it from previous studies that heterogeneously combined multiracial populations with different treatment effects due to various biological and genetic profiles.^57^^,^^58^ Second, compared with previous studies, this investigation included recent studies with new treatment regimens and lower follow-up LDL-C levels, which provides evidence to explore the effects of LDL-C <55 mg/dL. Third, this study not only analyzed the impacts of LLT on PAV, but also on FCT and LCBI, which provides us with a comprehensive understanding of the influence of LLT on CAP regression and stabilization. Fourth, this study used meticulous meta-regression analysis to investigate factors associated with the impacts of LLT on CAP, which provides evidence for future precise treatments.

### Study limitations

First, differences in patient populations, methods, follow-up duration, and different imaging modalities might contribute to heterogeneity, which was also observed in similar research.^59^^,^^60^ To account for this limitation, we used a more robust and conservative assessment of the pooled effect size through a random-effects model. We also performed sensitivity analyses by removing a specific study that was the main source of significant heterogeneity, which led to more consistent results with a reduced heterogeneity. Furthermore, high-impact studies with similar methodologies have been reported in the literature,^19^^,^^20^ supporting our study methodology. Second, with the limited number of studies, some subgroup analyses showed a wider estimation of treatment effects for some of the results. Third, because of limited resources, this study did not perform individual patient data meta-analysis to investigate whether other factors were associated with the impacts of LLT on CAP. Fourth, adherence to the principles of evidence-based medicine dictates that such data cannot be extrapolated to non-Asian races or ethnicities without further research. Fifth, our study did not evaluate clinical outcomes. Further studies are warranted to confirm association between clinical outcomes and plaque morphology changes by LLT in East Asian patients.

## Conclusions

LLT appears to have beneficial effects on CAP stability and regression in East Asian patients with established ASCVD or increased risks of ASCVD. Intensive LLT with a lower target LDL-C level, especially <55 mg/dL, seems to be advantageous for treating atherosclerosis in East Asian patients. Multicenter, large-sample, prospective studies are needed to further validate our findings.

## Funding Support and Author Disclosures

This work is supported by Amgen K.K. Dr Tsujita has received remuneration for lectures from Abbott, Amgen, Bayer, Daiichi-Sankyo, Kowa, Boehringer Ingelheim, Novartis, Otsuka, Pfizer, Takeda, and TERUMO; trust research/joint research funds from Bayer, Bristol-Myers, Daiichi-Sankyo, MOCHIDA, EA Pharma, TAUNS Laboratories, Novo Nordisk, and PRA Health Sciences; and scholarships from Abbott, ITI, Boehringer Ingelheim, Otsuka, and Boston Scientific. Dr Tsujita is also affiliated with a department that is endowed by Abbott, Boston Scientific, Fides-one, GM Medical, ITI, Kaneka Medix, NIPRO, TERUMO, Philips, Getinge Group, Orbusneich Medical, BIOTRONIK, Fukuda Denshi, Japan Lifeline, Medtronic, and Boehringer Ingelheim. All other authors have reported that they have no relationships relevant to the contents of this paper to disclose.
