# Supplementary material for: The Effect of Lipid-Lowering Therapy on Coronary Artery Plaque in East Asia Population
Source: JACC Asia. 2025 Jul 15;5(8):1032–47. doi: 10.1016/j.jacasi.2025.05.016 (PMC12426849; doi:10.1016/j.jacasi.2025.05.016)
Supplement: Supplemental Material [file mmc1.docx]

Contents

[Supplementary File 1 2](#_Toc188877019)

[Supplementary File 2 13](#_Toc188877020)

[Supplementary Figures 19](#_Toc188877021)

[Supplementary Tables 34](#_Toc188877022)

# **Supplementary File 1**

**Supplementary search strategy**

**PubMed**

1. "Hydroxymethylglutaryl-CoA Reductase Inhibitors" [Pharmacological Action] OR "Hydroxymethylglutaryl-CoA Reductase Inhibitors"[Mesh] OR lipid-lowering*[tw] OR cholesterol absorption inhibitor*[tw] OR Statin* OR "HMG-CoA Reductase Inhibitor*"[tw] OR "Hydroxymethylglutaryl-CoA Reductase Inhibitor*"[tw] OR "Hydroxymethylglutaryl-Coenzyme-A Inhibitor*"[tw] OR Hydroxymethylglutaryl-CoA Inhibitor*[tw] OR atorvastatin[tw] OR cerivastatin[tw] OR bervastatin[tw] OR compactin[tw] OR fluindostatin[tw] OR mevinolin[tw] OR pitavastatin[tw] OR pravastatin[tw] OR "risedronic acid*"[tw] OR rosuvastatin[tw] OR simvastatin[tw] OR vytorin[tw] OR rosuzet[tw] OR roszet[tw] OR lovastatin[tw]
2. "Fibric Acids"[Mesh] OR "Fibric Acid*"[tw] OR Fibrate*[tw] OR Bezafibrate[tw] OR bezalip[tw] OR Clofenapate[tw] OR "Clofibric Acid*"[tw] OR Clofibrate[tw] OR Fenofibrate[tw] OR Gemfibrozil[tw] OR atromid[tw] OR ciprofibrate[tw] OR pemafibrate[tw]
3. "Ezetimibe"[Mesh] OR Ezetimibe[tw] OR "Colesevelam Hydrochloride"[Mesh] OR Colesevelam[tw] OR torcetrapib[tw] OR avasimibe[tw] OR implitapide[tw] OR "PCSK9 inhibitor*"[tw] OR evolocumab[tw] OR "amg 145"[tw] OR alirocumab[tw] OR "regn 727"[tw] OR "sar 236553"[tw] OR frovocimab[tw] OR "ly 3015014"[tw] OR inclisiran[tw] OR "alnpcssc"[tw] OR bile acid sequestrant*[tw]
4. #1 OR #2 OR #3
5. "Plaque, Atherosclerotic"[Mesh] OR "Atherosclerosis"[Mesh] OR "Ultrasonography, Interventional"[Mesh] OR "Tomography, Optical Coherence"[Mesh] OR "Spectroscopy, Near-Infrared"[Mesh] OR "Computed Tomography Angiography"[Mesh] OR "Magnetic Resonance Angiography"[Mesh] OR Plaque*[tw] OR Arterial Fatty Streak*[tw] OR Fibroatheroma*[tw] OR Atheroma*[tw] OR atherosclero*[tw] OR Atherogenes*[tw] OR atheros*[tw] OR atheroscleos*[tw] OR scleratheroma*[tw] OR intravascular ultrasound*[tw] OR IVUS[tw] OR optical interference tomograph*[tw] OR optical coherence tomograph*[tw] OR OCT[tw] OR near-infrared spectro*[tw] OR NIRS[tw] OR N-IR spectro*[tw] OR near infra-red spectro*[tw] OR near IR spectro*[tw] OR NIR spectro*[tw] OR computed tomography angiograph*[tw] OR CT angiograph*[tw] OR computed tomographic angiograph*[tw] OR CTA[tw] OR magnetic resonance angiograph*[tw] OR MR angiograph*[tw] OR MRI angiograph*[tw] OR NMR angiograph*[tw] OR MRA[tw]
6. #4 AND #5
7. "Asia, Eastern"[Mesh] OR Japan OR Japanese OR Tokyo OR Hokkaido OR Osaka OR Kyoto OR Yokohama OR Nagoya OR Kobe OR Fukuoka OR Sapporo OR Sendai OR Hiroshima OR Korea OR Korean OR Seoul OR Sejong OR Jeju OR Suwon OR Busan OR Daegu OR Incheon OR Gwangju OR Daejeon OR ulsan OR China OR Chinese OR Taiwan OR Taiwanese OR "Hong kong" OR Hongkong OR Macau OR Macao OR Beijing OR Shanghai OR Tianjin OR Chongqing OR "Inner Mongolia" OR Tibet OR Guangxi OR Sinkiang OR Ningxia OR Xinjiang OR Hebei OR Shanxi OR Liaoning OR Jilin OR Heilongjiang OR Jiangsu OR Zhejiang OR Anhui OR Fujian OR Jiangxi OR Shandong OR Henan OR Hubei OR Hunan OR Guangdong OR Hainan OR Sichuan OR Guizhou OR Yunnan OR Shaanxi OR Gansu OR Qinghai OR Mongolia* OR "Ulan Bator" OR Ulaanbaatar OR Erdent OR Darkhan OR Choybalsan OR Ondorhaan OR "North Korea" OR Pyongyang OR Kaesong OR Sinuiju OR Hamhung
8. #6 AND #7
9. ("controlled clinical trial"[pt] OR "Controlled Clinical Trials as Topic"[MeSH] OR "Random Allocation"[MeSH] OR "Double-Blind Method"[MeSH] OR "single-blind method"[MeSH] OR "Control Groups"[MeSH] OR "cross-over studies"[MeSH] OR random*[tiab] OR placebo[tiab] OR trial[tiab] OR groups[tiab] OR crossover[tiab] OR cross-over[tiab]) NOT ("Animals"[Mesh] NOT ("Humans"[Mesh] AND "Animals"[Mesh])) 3,662,915
10. ("Observational Study"[pt] OR "Observational Studies as Topic"[Mesh] OR "Cohort Studies"[Mesh] OR "Case-Control Studies"[Mesh] OR "Cross-Sectional Studies"[Mesh] OR Observational Stud*[tiab] OR Cohort[tiab] OR "Follow-Up"[tiab] OR Longitudinal*[tiab] OR Prospectiv*[tiab] OR Retrospectiv*[tiab] OR "Case-Control"[tiab] OR "Cross-Sectional"[tiab] OR "case series"[tiab] OR "single arm"[tiab]) NOT ("Animals"[Mesh] NOT ("Humans"[Mesh] AND "Animals"[Mesh]))
11. #8 AND (#9 OR #10)

**Embase**

1. 'hydroxymethylglutaryl coenzyme A reductase inhibitor'/exp OR ("lipid-lowering*" OR "cholesterol absorption inhibitor*" OR Statin* OR "HMG-CoA Reductase Inhibitor*" OR "Hydroxymethylglutaryl-CoA Reductase Inhibitor*" OR "Hydroxymethylglutaryl-Coenzyme-A Inhibitor*" OR "Hydroxymethylglutaryl-CoA Inhibitor*" OR atorvastatin OR cerivastatin OR bervastatin OR compactin OR fluindostatin OR mevinolin OR pitavastatin OR pravastatin OR "risedronic acid*" OR rosuvastatin OR simvastatin OR vytorin OR rosuzet OR roszet OR lovastatin):ab,ti,kw
2. 'fibric acid derivative'/exp OR ("Fibric Acid*" OR Fibrate* OR Bezafibrate OR bezalip OR Clofenapate OR "Clofibric Acid*" OR Clofibrate OR Fenofibrate OR Gemfibrozil OR atromid OR ciprofibrate OR pemafibrate):ab,ti,kw 41,630
3. 'ezetimibe'/exp OR 'colesevelam'/exp OR (Ezetimibe OR Colesevelam OR torcetrapib OR avasimibe OR implitapide OR "PCSK9 inhibitor*" OR evolocumab OR "amg 145" OR alirocumab OR "regn 727" OR "sar 236553" OR frovocimab OR "ly 3015014" OR inclisiran OR "alnpcssc" OR "bile acid sequestrant*"):ab,ti,kw
4. #1 OR #2 OR #3
5. 'atherosclerotic plaque'/exp OR 'atherosclerosis'/exp OR 'intravascular ultrasound'/exp OR 'optical coherence tomography'/exp OR 'near infrared spectroscopy'/exp OR 'computed tomographic angiography'/exp OR 'magnetic resonance angiography'/exp OR (Plaque* OR "Arterial Fatty Streak*" OR Fibroatheroma* OR Atheroma* OR atherosclero* OR Atherogenes* OR atheros* OR atheroscleos* OR scleratheroma* OR (intravascular NEAR/3 ultrasound) OR IVUS OR (optical NEAR/3 tomograph*) OR OCT OR (("near-infrared" OR "N-IR" OR "near infra-red" OR "near IR" OR NIR) NEAR/3 spectro*) OR NIRS OR (("computed tomograph*" OR CT) NEAR/3 angiograph*) OR CTA OR (("magnetic resonance" OR MR OR MRI OR NMR) NEAR/3 angiograph*) OR MRA):ab,ti,kw
6. #4 AND #5
7. 'China'/exp OR 'Japan'/exp OR 'Korea'/exp OR 'Mongolia'/exp OR 'Taiwan'/exp OR (Japan OR Japanese OR Tokyo OR Hokkaido OR Osaka OR Kyoto OR Yokohama OR Nagoya OR Kobe OR Fukuoka OR Sapporo OR Sendai OR Hiroshima OR Korea OR Korean OR Seoul OR Sejong OR Jeju OR Suwon OR Busan OR Daegu OR Incheon OR Gwangju OR Daejeon OR ulsan OR China OR Chinese OR Taiwan OR Taiwanese OR "Hong kong" OR Hongkong OR Macau OR Macao OR Beijing OR Shanghai OR Tianjin OR Chongqing OR "Inner Mongolia" OR Tibet OR Guangxi OR Sinkiang OR Ningxia OR Xinjiang OR Hebei OR Shanxi OR Liaoning OR Jilin OR Heilongjiang OR Jiangsu OR Zhejiang OR Anhui OR Fujian OR Jiangxi OR Shandong OR Henan OR Hubei OR Hunan OR Guangdong OR Hainan OR Sichuan OR Guizhou OR Yunnan OR Shaanxi OR Gansu OR Qinghai OR Mongolia* OR "Ulan Bator" OR Ulaanbaatar OR Erdent OR Darkhan OR Choybalsan OR Ondorhaan OR "North Korea" OR Pyongyang OR Kaesong OR Sinuiju OR Hamhung):ti,ab,ad,ff
8. #7 AND #8
9. ('controlled clinical trial'/exp OR 'Controlled Clinical Trial (Topic)'/exp OR 'double blind procedure'/de OR 'control group'/de OR 'crossover procedure'/de OR 'single blind procedure'/de OR 'triple blind procedure'/de OR 'placebo'/de OR 'randomization'/exp OR (random* OR trial OR groups OR placebo* OR crossover OR "cross-over"):ab,ti,kw) NOT (('nonhuman'/exp OR 'animal'/exp) NOT 'human'/exp)
10. ('observational study'/exp OR 'cohort analysis'/exp OR 'cross-sectional study'/exp OR 'case control study'/exp OR ("observational stud*" OR Cohort OR "Follow-Up" OR Longitudinal* OR Prospectiv* OR Retrospectiv* OR "Case-Control" OR "Cross-Sectional" OR "case-series" OR "single arm"):ab,ti,kw) NOT (('nonhuman'/exp OR 'animal'/exp) NOT 'human'/exp)
11. #8 AND (#9 OR #10)

**Wos**

1. TS=("lipid-lowering*" OR "cholesterol absorption inhibitor*" OR Statin* OR "HMG-CoA Reductase Inhibitor*" OR "Hydroxymethylglutaryl-CoA Reductase Inhibitor*" OR "Hydroxymethylglutaryl-Coenzyme-A Inhibitor*" OR "Hydroxymethylglutaryl-CoA Inhibitor*" OR atorvastatin OR cerivastatin OR bervastatin OR compactin OR fluindostatin OR mevinolin OR pitavastatin OR pravastatin OR "risedronic acid*" OR rosuvastatin OR simvastatin OR vytorin OR rosuzet OR roszet OR lovastatin)
2. TS=("Fibric Acid*" OR Fibrate* OR Bezafibrate OR bezalip OR Clofenapate OR "Clofibric Acid*" OR Clofibrate OR Fenofibrate OR Gemfibrozil OR atromid OR ciprofibrate OR pemafibrate)
3. TS=(Ezetimibe OR Colesevelam OR torcetrapib OR avasimibe OR implitapide OR "PCSK9 inhibitor*" OR evolocumab OR "amg 145" OR alirocumab OR "regn 727" OR "sar 236553" OR frovocimab OR "ly 3015014" OR inclisiran OR "alnpcssc" OR "bile acid sequestrant*")
4. #1 OR #2 OR #3
5. TS=(Plaque* OR "Arterial Fatty Streak*" OR Fibroatheroma* OR Atheroma* OR atherosclero* OR Atherogenes* OR atheros* OR atheroscleos* OR scleratheroma* OR (intravascular NEAR/3 ultrasound) OR IVUS OR (optical NEAR/3 tomograph*) OR OCT OR (("near-infrared" OR "N-IR" OR "near infra-red" OR "near IR" OR NIR) NEAR/3 spectro*) OR NIRS OR (("computed tomograph*" OR CT) NEAR/3 angiograph*) OR CTA OR (("magnetic resonance" OR MR OR MRI OR NMR) NEAR/3 angiograph*) OR MRA)
6. #4 AND #5
7. CU=(JAPAN or PEOPLES R CHINA or SOUTH KOREA or TAIWAN or MONGOLIA or NORTH KOREA or HONG KONG) OR TS=(Japan OR Japanese OR Tokyo OR Hokkaido OR Osaka OR Kyoto OR Yokohama OR Nagoya OR Kobe OR Fukuoka OR Sapporo OR Sendai OR Hiroshima OR Korea OR Korean OR Seoul OR Sejong OR Jeju OR Suwon OR Busan OR Daegu OR Incheon OR Gwangju OR Daejeon OR ulsan OR China OR Chinese OR Taiwan OR Taiwanese OR "Hong kong" OR Hongkong OR Macau OR Macao OR Beijing OR Shanghai OR Tianjin OR Chongqing OR "Inner Mongolia" OR Tibet OR Guangxi OR Sinkiang OR Ningxia OR Xinjiang OR Hebei OR Shanxi OR Liaoning OR Jilin OR Heilongjiang OR Jiangsu OR Zhejiang OR Anhui OR Fujian OR Jiangxi OR Shandong OR Henan OR Hubei OR Hunan OR Guangdong OR Hainan OR Sichuan OR Guizhou OR Yunnan OR Shaanxi OR Gansu OR Qinghai OR Mongolia* OR "Ulan Bator" OR Ulaanbaatar OR Erdent OR Darkhan OR Choybalsan OR Ondorhaan OR "North Korea" OR Pyongyang OR Kaesong OR Sinuiju OR Hamhung) OR AD=(Japan OR Japanese OR Tokyo OR Hokkaido OR Osaka OR Kyoto OR Yokohama OR Nagoya OR Kobe OR Fukuoka OR Sapporo OR Sendai OR Hiroshima OR Korea OR Korean OR Seoul OR Sejong OR Jeju OR Suwon OR Busan OR Daegu OR Incheon OR Gwangju OR Daejeon OR ulsan OR China OR Chinese OR Taiwan OR Taiwanese OR "Hong kong" OR Hongkong OR Macau OR Macao OR Beijing OR Shanghai OR Tianjin OR Chongqing OR "Inner Mongolia" OR Tibet OR Guangxi OR Sinkiang OR Ningxia OR Xinjiang OR Hebei OR Shanxi OR Liaoning OR Jilin OR Heilongjiang OR Jiangsu OR Zhejiang OR Anhui OR Fujian OR Jiangxi OR Shandong OR Henan OR Hubei OR Hunan OR Guangdong OR Hainan OR Sichuan OR Guizhou OR Yunnan OR Shaanxi OR Gansu OR Qinghai OR Mongolia* OR "Ulan Bator" OR Ulaanbaatar OR Erdent OR Darkhan OR Choybalsan OR Ondorhaan OR "North Korea" OR Pyongyang OR Kaesong OR Sinuiju OR Hamhung)
8. #7 AND #8
9. (TS=(((controlled OR Random*) NEAR/5 (Trial OR study)) OR "Random Allocation*" OR Randomization* OR "Double Blind" OR "single blind" OR "Control Group*" OR "Controlled Group*" OR "cross over" OR placebo* OR crossover OR random*) OR TI=("trial" OR "RCT" OR "groups" OR "group"))
10. TS=("case control" OR family OR longitudinal* OR retrospect* OR prospect* OR cohort OR "follow up" OR observational OR epidemiological OR "cross sectional" OR "cross-sectional" OR "single arm")
11. #8 AND (#9 OR #10)
12. TI=(veterinary OR rabbit* OR animal* OR mouse OR mice OR rodent* OR rat OR rats OR pig OR pigs OR porcine OR horse OR horses OR equine OR cow OR cows OR bovine OR goat OR goats OR sheep OR ovine OR canine OR dog OR dogs OR feline OR cat OR cats OR review OR "case report" OR "a case")
13. #11 NOT #12

**Cochrane**

#1 MeSH descriptor: [Hydroxymethylglutaryl-CoA Reductase Inhibitors] explode all trees 4500

#2 MeSH descriptor: [Fibric Acids] explode all trees

#3 MeSH descriptor: [Ezetimibe] explode all trees

#4 MeSH descriptor: [Colesevelam Hydrochloride] explode all trees

#5 ("lipid-lowering*" OR "cholesterol absorption inhibitor*" OR Statin* OR "HMG-CoA Reductase Inhibitor*" OR "Hydroxymethylglutaryl-CoA Reductase Inhibitor*" OR "Hydroxymethylglutaryl-Coenzyme-A Inhibitor*" OR "Hydroxymethylglutaryl-CoA Inhibitor*" OR atorvastatin OR cerivastatin OR bervastatin OR compactin OR fluindostatin OR mevinolin OR pitavastatin OR pravastatin OR "risedronic acid*" OR rosuvastatin OR simvastatin OR vytorin OR rosuzet OR roszet OR lovastatin):ti,ab,kw

#6 ("Fibric Acid*" OR Fibrate* OR Bezafibrate OR bezalip OR Clofenapate OR "Clofibric Acid*" OR Clofibrate OR Fenofibrate OR Gemfibrozil OR atromid OR ciprofibrate OR pemafibrate):ti,ab,kw

#7 (Ezetimibe OR Colesevelam OR torcetrapib OR avasimibe OR implitapide OR "PCSK9 inhibitor*" OR evolocumab OR "amg 145" OR alirocumab OR "regn 727" OR "sar 236553" OR frovocimab OR "ly 3015014" OR inclisiran OR "alnpcssc" OR "bile acid sequestrant*"):ti,ab,kw

#8 #1 OR #2 OR #3 OR #4 OR #5 OR #6 OR #7

#9 MeSH descriptor: [Plaque, Atherosclerotic] explode all trees

#10 MeSH descriptor: [Atherosclerosis] explode all trees

#11 MeSH descriptor: [Ultrasonography, Interventional] explode all trees

#12 MeSH descriptor: [Tomography, Optical Coherence] explode all trees

#13 MeSH descriptor: [Spectroscopy, Near-Infrared] explode all trees

#14 MeSH descriptor: [Computed Tomography Angiography] explode all trees

#15 MeSH descriptor: [Magnetic Resonance Angiography] explode all trees

#16 (Plaque* OR "Arterial Fatty Streak*" OR Fibroatheroma* OR Atheroma* OR atherosclero* OR Atherogenes* OR atheros* OR atheroscleos* OR scleratheroma* OR (intravascular NEAR/3 ultrasound) OR IVUS OR (optical NEAR/3 tomograph*) OR OCT OR (("near-infrared" OR "N-IR" OR "near infra-red" OR "near IR" OR NIR) NEAR/3 spectro*) OR NIRS OR (("computed tomograph*" OR CT) NEAR/3 angiograph*) OR CTA OR (("magnetic resonance" OR MR OR MRI OR NMR) NEAR/3 angiograph*) OR MRA):ti,ab,kw

#17 #9 OR #10 OR #11 OR #12 OR #13 OR #14 OR #15 OR #16

#18 #8 AND #17

#19 MeSH descriptor: [Asia, Eastern] explode all trees

#20 Japan OR Japanese OR Tokyo OR Hokkaido OR Osaka OR Kyoto OR Yokohama OR Nagoya OR Kobe OR Fukuoka OR Sapporo OR Sendai OR Hiroshima OR Korea OR Korean OR Seoul OR Sejong OR Jeju OR Suwon OR Busan OR Daegu OR Incheon OR Gwangju OR Daejeon OR ulsan OR China OR Chinese OR Taiwan OR Taiwanese OR "Hong kong" OR Hongkong OR Macau OR Macao OR Beijing OR Shanghai OR Tianjin OR Chongqing OR "Inner Mongolia" OR Tibet OR Guangxi OR Sinkiang OR Ningxia OR Xinjiang OR Hebei OR Shanxi OR Liaoning OR Jilin OR Heilongjiang OR Jiangsu OR Zhejiang OR Anhui OR Fujian OR Jiangxi OR Shandong OR Henan OR Hubei OR Hunan OR Guangdong OR Hainan OR Sichuan OR Guizhou OR Yunnan OR Shaanxi OR Gansu OR Qinghai OR Mongolia* OR "Ulan Bator" OR Ulaanbaatar OR Erdent OR Darkhan OR Choybalsan OR Ondorhaan OR "North Korea" OR Pyongyang OR Kaesong OR Sinuiju OR Hamhung

#21 #19 OR #20

#22 #18 AND #21

**Supplementary list of included studies**

1. Ako, J., et al., *Effect of Alirocumab on Coronary Atheroma Volume in Japanese Patients With Acute Coronary Syndrome　- The ODYSSEY J-IVUS Trial.* Circulation journal : official journal of the Japanese Circulation Society, 2019. 83(10): p. 2025-2033.

2. Dong, N., et al., *Statin-induced improvements in vulnerable plaques are attenuated in poorly controlled diabetic patients with coronary atherosclerosis disease: a serial optical coherence tomography analysis.* Acta diabetologica, 2016. 53(6): p. 999-1008.

3. Gao, F., et al., *Effect of alirocumab on coronary plaque in patients with coronary artery disease assessed by optical coherence tomography.* Lipids in health and disease, 2021. 20(1): p. 106.

4. Guo, S., et al., *Effects of atorvastatin on serum lipids, serum inflammation and plaque morphology in patients with stable atherosclerotic plaques.* Experimental and therapeutic medicine, 2012. 4(6): p. 1069-1074.

5. Habara, M., et al., *Impact on Optical Coherence Tomographic Coronary Findings of Fluvastatin Alone Versus Fluvastatin + Ezetimibe.* American Journal of Cardiology, 2014. 113(4): p. 580-587.

6. Hattori, K., et al., *Impact of statin therapy on plaque characteristics as assessed by serial OCT, grayscale and integrated backscatter-IVUS.* JACC. Cardiovascular imaging, 2012. 5(2): p. 169-77.

7. Hibi, K., et al., *Effects of Ezetimibe-Statin Combination Therapy on Coronary Atherosclerosis in Acute Coronary Syndrome.* Circulation journal : official journal of the Japanese Circulation Society, 2018. 82(3): p. 757-766.

8. Hiro, T., et al., *Effect of early intensive statin therapy on regression of coronary atherosclerosis in patients with acute coronary syndrome: Rationale for lower cholesterol target in diabetic patients: Subanalysis of JAPAN-ACS study.* Journal of the American College of Cardiology, 2009. 53(10): p. A330.

9. Hong, M.K., et al., *Effects of statin treatments on coronary plaques assessed by volumetric virtual histology intravascular ultrasound analysis.* JACC. Cardiovascular interventions, 2009. 2(7): p. 679-88.

10. Hong, Y.J., et al., *Effect of pitavastatin treatment on changes of plaque volume and composition according to the reduction of high-sensitivity C-reactive protein levels.* Journal of cardiology, 2012. 60(4): p. 277-82.

11. Hong, Y.J., et al., *The effects of rosuvastatin on plaque regression in patients who have a mild to moderate degree of coronary stenosis with vulnerable plaque.* Korean circulation journal, 2008. 38(7): p. 366-373.

12. Hong, Y.J., et al., *Comparison of effects of rosuvastatin and atorvastatin on plaque regression in Korean patients with untreated intermediate coronary stenosis.* Circulation journal : official journal of the Japanese Circulation Society, 2011. 75(2): p. 398-406.

13. Hou, J., et al., *Comparison of Intensive Versus Moderate Lipid-Lowering Therapy on Fibrous Cap and Atheroma Volume of Coronary Lipid-Rich Plaque Using Serial Optical Coherence Tomography and Intravascular Ultrasound Imaging.* The American journal of cardiology, 2016. 117(5): p. 800-6.

14. Ishikawa, Y., et al., *Impact of water- and lipid-soluble statins on nonculprit lesions in patients with acute coronary syndrome: A prospective randomized trial.* International heart journal, 2018. 59(1): p. 27-34.

15. Kashiyama, K., et al., *Coronary plaque progression of non-culprit lesions after culprit percutaneous coronary intervention in patients with moderate to advanced chronic kidney disease: intravascular ultrasound and integrated backscatter intravascular ultrasound study.* The international journal of cardiovascular imaging, 2015. 31(5): p. 935-45.

16. Kodama, K., et al., *Stabilization and regression of coronary plaques treated with pitavastatin proven by angioscopy and intravascular ultrasound--the TOGETHAR trial.* Circulation journal : official journal of the Japanese Circulation Society, 2010. 74(9): p. 1922-8.

17. Komukai, K., et al., *Effect of atorvastatin therapy on fibrous cap thickness in coronary atherosclerotic plaque as assessed by optical coherence tomography: the EASY-FIT study.* Journal of the American College of Cardiology, 2014. 64(21): p. 2207-17.

18. Lee, C.H., et al., *Effect of Atorvastatin on Serial Changes in Coronary Physiology and Plaque Parameters.* JACC. Asia, 2022. 2(6): p. 691-703.

19. Lee, C.W., et al., *Comparison of effects of atorvastatin (20 mg) versus rosuvastatin (10 mg) therapy on mild coronary atherosclerotic plaques (from the ARTMAP trial).* The American journal of cardiology, 2012. 109(12): p. 1700-4.

20. Lee, J.H., et al., *Early Effects of Intensive Lipid-Lowering Treatment on Plaque Characteristics Assessed by Virtual Histology Intravascular Ultrasound.* Yonsei medical journal, 2016. 57(5): p. 1087-94.

21. Lee, S.W., et al., *Virtual histology findings and effects of varying doses of atorvastatin on coronary plaque volume and composition in statin-naive patients: the VENUS study.* Circulation journal : official journal of the Japanese Circulation Society, 2012. 76(11): p. 2662-72.

22. Masuda, J., et al., *Effect of Combination therapy of Ezetimibe and Rosuvastatin on Regression of Coronary Atherosclerosis in Patients with Coronary Artery Disease.* International heart journal, 2015. 56(3): p. 278-285.

23. Matsushita, K., et al., *Effects of 4 Statins on Regression of Coronary Plaque in Acute Coronary Syndrome.* Circulation journal : official journal of the Japanese Circulation Society, 2016. 80(7): p. 1634-43.

24. Meng, P.N., et al., *Morphologies and composition changes in nonculprit subclinical atherosclerosis in diabetic versus nondiabetic patients with acute coronary syndrome who underwent long-term statin therapy.* Scientific reports, 2023. 13(1): p. 5338.

25. Meng, P.N., et al., *Intensive statin versus low-dose statin + ezetimibe treatment for fibrous cap thickness of coronary vulnerable plaques.* Chinese medical journal, 2020. 133(20): p. 2415-2421.

26. Nakajima, N., et al., *Effect of combination of ezetimibe and a statin on coronary plaque regression in patients with acute coronary syndrome: ZEUS trial (eZEtimibe Ultrasound Study).* IJC Metabolic and Endocrine, 2014. 3: p. 8-13.

27. Nishiguchi, T., et al., *Effect of Early Pitavastatin Therapy on Coronary Fibrous-Cap Thickness Assessed by Optical Coherence Tomography in Patients With Acute Coronary Syndrome: The ESCORT Study.* JACC. Cardiovascular imaging, 2018. 11(6): p. 829-838.

28. Nozue, T., et al., *Statin treatment for coronary artery plaque composition based on intravascular ultrasound radiofrequency data analysis.* American heart journal, 2012. 163(2): p. 191-9.e1.

29. Oh, P.C., et al., *Effect of Atorvastatin (10 mg) and Ezetimibe (10 mg) Combination Compared to Atorvastatin (40 mg) Alone on Coronary Atherosclerosis.* The American journal of cardiology, 2021. 154: p. 22-28.

30. Okazaki, S., et al., *Early statin treatment in patients with acute coronary syndrome: demonstration of the beneficial effect on atherosclerotic lesions by serial volumetric intravascular ultrasound analysis during half a year after coronary event: the ESTABLISH Study.* Circulation, 2004. 110(9): p. 1061-8.

31. Ota, H., et al., *Clinical impact of PCSK9 inhibitor on stabilization and regression of lipid-rich coronary plaques: a near-infrared spectroscopy study.* European heart journal. Cardiovascular Imaging, 2022. 23(2): p. 217-228.

32. Otagiri, K., et al., *Early intervention with rosuvastatin decreases the lipid components of the plaque in acute coronary syndrome: analysis using integrated backscatter IVUS (ELAN study).* Circulation journal : official journal of the Japanese Circulation Society, 2011. 75(3): p. 633-41.

33. Park, S.J., et al., *Effect of Statin Treatment on Modifying Plaque Composition: A Double-Blind, Randomized Study.* Journal of the American College of Cardiology, 2016. 67(15): p. 1772-1783.

34. Shin, E.S., et al., *Effect of statins on coronary bifurcation atherosclerosis: an intravascular ultrasound virtual histology study.* The international journal of cardiovascular imaging, 2012. 28(7): p. 1643-52.

35. Sugizaki, Y., et al., *Adding Alirocumab to Rosuvastatin Helps Reduce the Vulnerability of Thin-Cap Fibroatheroma: An ALTAIR Trial Report.* JACC: Cardiovascular Imaging, 2020. 13(6): p. 1452-1454.

36. Takayama, T., et al., *Effect of rosuvastatin on coronary atheroma in stable coronary artery disease: multicenter coronary atherosclerosis study measuring effects of rosuvastatin using intravascular ultrasound in Japanese subjects (COSMOS).* Circulation journal : official journal of the Japanese Circulation Society, 2009. 73(11): p. 2110-7.

37. Takayama, T., et al., *Comparison of the Effect of Rosuvastatin 2.5 mg vs 20 mg on Coronary Plaque Determined by Angioscopy and Intravascular Ultrasound in Japanese With Stable Angina Pectoris (from the Aggressive Lipid-Lowering Treatment Approach Using Intensive Rosuvastatin for Vulnerable Coronary Artery Plaque [ALTAIR] Randomized Trial).* The American journal of cardiology, 2016. 117(8): p. 1206-12.

38. Tani, S., et al., *Coronary plaque regression and lifestyle modification in patients treated with pravastatin. - Assessment mainly by daily aerobic exercise and an increase in the serum level of high-density lipoprotein cholesterol.* Circulation journal : official journal of the Japanese Circulation Society, 2010. 74(5): p. 954-61.

39. Tani, S., et al., *Effect of pravastatin on malondialdehyde-modified low-density lipoprotein levels and coronary plaque regression as determined by three-dimensional intravascular ultrasound.* The American journal of cardiology, 2005. 96(8): p. 1089-94.

40. Thondapu, V., et al., *Comparison of Rosuvastatin Versus Atorvastatin for Coronary Plaque Stabilization.* The American journal of cardiology, 2019. 123(10): p. 1565-1571.

41. Tsujita, K., et al., *Impact of Dual Lipid-Lowering Strategy With Ezetimibe and Atorvastatin on Coronary Plaque Regression in Patients With Percutaneous Coronary Intervention: The Multicenter Randomized Controlled PRECISE-IVUS Trial.* Journal of the American College of Cardiology, 2015. 66(5): p. 495-507.

42. Ueda, Y., et al., *Effect of Ezetimibe on Stabilization and Regression of Intracoronary Plaque　- The ZIPANGU Study.* Circulation journal : official journal of the Japanese Circulation Society, 2017. 81(11): p. 1611-1619.

43. Wang, X.F., C.Z. Lu, and X. Chen, *[Effects of statins on coronary atherosclerotic plaque in patients with coronary heart disease and type 2 diabetes with mild elevated LDL-C].* Zhonghua xin xue guan bing za zhi, 2009. 37(4): p. 339-42.

44. Xu, M., et al., *Is the effect of atorvastatin 60 mg on stabilization of lipid-rich plaque equivalent to that of rosuvastatin 10 mg? A serial optical coherence tomography combined with intravascular ultrasound imaging.* Catheterization and cardiovascular interventions : official journal of the Society for Cardiac Angiography & Interventions, 2021. 97 Suppl 2: p. 1097-1107.

45. Yano, H., S. Horinaka, and T. Ishimitsu, *Effect of evolocumab therapy on coronary fibrous cap thickness assessed by optical coherence tomography in patients with acute coronary syndrome.* Journal of cardiology, 2020. 75(3): p. 289-295.

46. Yokoyama, M., et al., *Plasma low-density lipoprotein reduction and structural effects on coronary atherosclerotic plaques by atorvastatin as clinically assessed with intravascular ultrasound radio-frequency signal analysis: a randomized prospective study.* American heart journal, 2005. 150(2): p. 287.

47. Zhang, X., et al., *Intensive-dose atorvastatin regimen halts progression of atherosclerotic plaques in new-onset unstable angina with borderline vulnerable plaque lesions.* Journal of cardiovascular pharmacology and therapeutics, 2013. 18(2): p. 119-25.

48. Zhu, Y., et al., *Impact of statins therapy on morphological changes in lipid-rich plaques stratified by 10-Year framingham risk score: A serial optical coherence tomography study.* Oncotarget, 2017. 8(16): p. 27401-27411.

# **Supplementary File 2**

**Baseline characteristics**

| **Study ID** | **Country** | **Study design** | **Detection Methods** | **Sample size** | **Patients (diseases)** | **Age (years, Mean ± SD)** | **Treatment duration** | **Drug-Dose (mg/day)** |
| --- | --- | --- | --- | --- | --- | --- | --- | --- |
| Ako 2019 | Japan | RCT | J-IVUS | 182 | Acute coronary syndrome | Standard of care: 60.5 ± 11.6; Alirocumab group: 61.8 ± 10.2 | 9 months | Alirocumab group: Atorvastatin 10 mg/20 mg/d, Rosuvastatin 5 mg/10mg/20 mg /d+Alirocumab75mg Q2W-150mg Q2W Standard of care: Atorvastatin 10 mg/20 mg/d, Rosuvastatin 5 mg/10mg/20 mg/d; |
| Chun 2021 | South Korea | RCT | NIRS-IVUS | 37 | Coronary artery disease | Atorvastatin+ ezetimibe: 56.3 ±7.1； Atorvastatin: 56.7 ± 8.4 | 12 months (± 4 weeks) | Atorvastatin 10 mg/d+ ezetimibe 10 mg/d ； Atorvastatin 40 mg/d |
| Dong 2016 | China | Cohort study | OCT | 75 | Coronary artery disease | 55.7 ± 9.6 | 12 months | NR |
| Gao 2021 | China | RCT | OCT | 61 | Coronary lesions | atorvastatin or rosuvastatin +alirocumab :61.3 ± 9.9  atorvastatin or rosuvastatin :61.3 ± 8.9 | 9 months | atorvastatin 20 mg/d or rosuvastatin 10 mg/d + alirocumab 75mg Q2W; atorvastatin 20 mg/d or rosuvastatin 10 mg/d |
| GUO 2012 | China | RCT | IVUS | 174 | Stable atherosclerotic plaques | Atorvastatin 10mg: 62.64 ± 12.00 Atorvastatin 20mg: 59.18 ± 8.48 Atorvastatin 40mg: 58.91 ± 12.90 Atorvastatin 80mg: 58.95 ± 9.68 | 6 months | Atorvastatin: 10mg/d Atorvastatin: 20mg/d Atorvastatin: 40mg/d Atorvastatin: 80mg/d |
| Habara 2014 | Japan | RCT | OCT | 63 | Coronary artery disease | Fluvastatin + Ezetimibe: 69.8 ± 7.8 Fluvastatin: 68.8±7.8 | 9 months | Fluvastatin 30mg/d + Ezetimibe 10mg/d; Fluvastatin 30mg/d |
| Hattori 2012 | Japan | case-control prospectively study | OCT+IVUS | 42 | Stable angina | Pitavastatin: 66 ± 7.8  Control group: 68 ± 6.2 | 9 months | Pitavastatin: 4mg/d Control group: diet therapy |
| Hibi 2018 | Japan | RCT | IVUS | 103 | Acute coronary syndrome | Pitavastatin + ezetimibe: 63±10 Pitavastatin: 63±12 | 10 months | Pitavastatin + ezetimibe: pitavastatin 2mg/d + ezetimibe 10mg/d Pitavastatin: 2mg/d |
| Hiro 2009 | Japan | RCT | IVUS | 252 | Acute coronary syndrome | Pitavastatin:62.5±11.5 Atorvastatin:62.4±10.6 | Pitavastatin: median (interquartile range)：9.3 (8.5-10.3) atorvastatin: median (interquartile range)：9.6 (8.6-10.5) | Pitavastatin: 4mg/d Atorvastatin:20mg/d |
| Hong 2008 | South Korea | RCT | IVUS | 30 | Coronary artery disease | Rosuvastatin 20:60 ± 8  Atorvastatin 40:62 ± 9 | 12 months | Rosuvastatin：20mg/d Atorvastatin：40mg/d |
| Hong 2009 | South Korea | RCT | IVUS | 100 | de novo no culprit + nontarget lesions | Simvastatin:58 ± 10 Rosuvastatin:59 ± 9 | 12 months | Simvastatin: 20mg/d Rosuvastatin: 10mg/d |
| Hong 2011 | South Korea | RCT | IVUS | 128 | intermediate coronary stenosis | Rosuvastatin: 59±10 Atorvastatin: 58±10 | 11 months | Rosuvastatin: 20mg/d Atorvastatin: 40mg/d |
| Hong 2012 | South Korea | Cohort study | IVUS | 94 | Acute myocardial infarction (AMI) | hs-CRP reduction <1 mg/dl:64 ± 12 hs-CRP reduction ≥1 mg/dl:63 ± 9 | NR | pitavastatin:2 mg/d |
| Hou 2015 | China | RCT | OCT+IVUS | 46 | coronary artery disease | atorvastatin(60mg):55±10 atorvastatin:(20mg):54±9.3 | 6/12 months | atorvastatin:60mg/d; atorvastatin:20mg/d |
| Ishikawa 2018 | Japan | RCT | IVUS | 35 | Acute Coronary Syndrome | Atorvastatin:57.4 ± 11.9  Rosuvastatin:65.9 ± 7.6 | Atorvastatin 10mg: 7.3 ± 1.6 months Rosuvastatin 2.5mg: 7.8 ± 2.3 months | Atorvastatin: 10mg/d Rosuvastatin: 2.5mg/d |
| Kashiyama 2015 | Japan | Cohort study | IB-IVUS | 113 | Chronic kidney disease +coronary artery disease | chronic kidney disease (CKD)-1: 61 ± 12; chronic kidney disease (CKD)-2: 70 ± 9; chronic kidney disease (CKD)-3: 77 ± 9; chronic kidney disease (CKD) 4-5: 69 ± 9 | 8 months | Statins (rosuvastatin, atorvastatin, pitavastatin or pravastatin) |
| Kodama 2010 | Japan | Cohort study | IVUS | 46 | patients with coronary plaque | 62.5±10.3 | 13 months | Pitavastatin 2mg/d |
| Komukai 2014 | Japan | RCT | OCT | 60 | unstable angina pectoris + dyslipidemia | Atorvastatin 20mg/d: median (interquartile range): 63 (58 - 73) Atorvastatin 5mg/d: median (interquartile range): 69 (58–74) | 12 months | Atorvastatin: 20mg/d; Atorvastatin: 5mg/d |
| Lee 2012-a | South Korea | Cohort study | IVUS | 271 | Coronary atherosclerotic plaques | Atorvastatin: 57.6 ± 7.6; Rosuvastatin: 55.3 ± 9.4 | 6 months | Atorvastatin: 20mg/d; Rosuvastatin: 10mg/d |
| Lee 2012-b | China | RCT | VH-IVUS | 39 | stable angina | Atorvastatin(10mg):65.05±9.99; Atorvastatin(40mg):63.70±9.80 | 6 months | Atorvastatin:10mg/d; Atorvastatin:40mg/d |
| Lee 2016 | South Korea | Cohort study | IVUS | 70 | Acute coronary syndrome | Pravastatin: 59.3±10.7 Ezetimibe/simvastatin: 60.9±10.9 | 3 months | Pravastatin: 36mg/d Ezetimibe/simvastatin: 34mg/d |
| Lee 2022 | South Korea | Single arm trial | IVUS | 95 | Coronary artery disease | 60.5 ± 8.9 | 12 months | Atorvastatin: 42.7mg/d |
| Masuda 2015 | Japan | RCT | IVUS | 40 | Coronary Artery Disease | Rosuvastatin:70.2±7.6 Rosuvastatin+ ezetimibe: 64.0±7.9 | 6 months | Rosuvastatin: 5mg/d Rosuvastatin+ ezetimibe: 10mg/d |
| Matsushita 2015 | Japan | RCT | IVUS | 102 | Acute coronary syndrome | 62.8±10.2 | 10 months | Atorvastatin 20mg/d or pitavastatin 4mg/d or Pravastatin 10mg/d or Fluvastatin 30mg/d |
| Meng 2020 | China | Cohort study | OCT | 53 | Acute coronary syndromes | Rosuvastatin or atorvastatin: 64.31 ± 8.53； rosuvastatin or atorvastatin + ezetimibe: 63.56 ± 7.50 | 12 months | rosuvastatin 15 - 20 mg/d or atorvastatin 30 - 40 mg/d ； rosuvastatin 5 - 10 mg/d or atorvastatin 10 - 20 mg/d + ezetimibe 10 mg/d |
| Meng 2023 | China | RCT | OCT | 114 | Acute coronary syndrome | Median (range) 65.00 (58.00, 71.00) | 12 months | rosuvastatin 10 mg/d; atorvastatin 20 mg/d |
| Nakajima 2014 | Japan | CCT | IVUS | 95 | Acute coronary syndromes | Atorvastatin: 60.7 ± 10.5 ;  Atorvastatin + ezetimibe: 63.7 ± 12.6 | 6 months | Atorvastatin: 20 mg/d; Atorvastatin + ezetimibe: atorvastatin 20 mg/d and ezetimibe 10 mg/d |
| Nishiguchi 2018 | Japan | RCT | OCT | 53 | Acute coronary syndromes | Median (range) Pitavastatin (Early Statin Group): 66 (63 - 71); Pitavastatin (Late Statin Group): 66 (62 - 74) | 1/9months | Pitavastatin (Early Statin Group): 4 mg/d from baseline; Pitavastatin (Late Statin Group): 4 mg/d from 3 weeks after the baseline |
| Nozue 2012 | Japan | Cohort study | VH-IVUS | 119 | Angina Pectoris | Pitavastatin: 66 ± 9; Pravastatin: 67 ± 11 | 8 months | Pitavastatin: 4 mg/d Pravastatin: 20 mg/d |
| Okazaki 2004 | Japan | RCT | IVUS | 70 | Acute coronary syndrome | 61.9±10.6 Control Group: 62.5±11.2 | 6 months | Atorvastatin: 20mg/d Control Group: NR |
| Ota 2022 | Japan | Cohort study | NIRS-IVUS | 53 | ACS or chronic coronary syndrome (CCS) | PCSK9i and statins :63.8 ± 13.4; statins :69.5 ± 10.0 | 12±3 months | PCSK9i and statins: evolocumab 140 mg or alirocumab 75 mg Q2W， statins :NR |
| Otagiri 2011 | Japan | Cohort study | IB-IVUS | 20 | Acute Coronary Syndrome | 70.5±8.9 | 6.2±1.2 months | Rosuvastatin: 5 mg/d |
| Park 2016 | South Korea | RCT | IVUS | 225 | coronary artery disease | 62.3±9.2 | 12 months | Rosuvastatin: 40mg/d; Rosuvastatin: 10mg/d |
| Shin 2011 | South Korea | RCT | VH-IVUS | 48 | Coronary bifurcation atherosclerosis | 61.3±8.5 | 12 months | Simvastatin: 20mg/d Rosuvastatin: 10mg/d |
| Sugizaki 2020 | Japan | RCT | OCT | 24 | Coronary artery disease | NR | 9 months | Alirocumab + Rosuvastatin: alirocumab 75 mg every 2 weeks and 10 mg/d rosuvastatin Rosuvastatin :10 mg/d |
| Takayama 2009 | Japan | CCT | IVUS | 126 | coronary artery disease | 62.6±7.7 | 19 months | rosuvastatin: 2.5-20mg/d |
| Takayama 2016 | Japan | RCT | IVUS | 37 | stable angina or silent myocardial ischemia | Rosuvastatin 20mg: 65.1±10.1  Rosuvastatin 2.5mg: 63.8±8.5 | 12 months | Rosuvastatin: 20mg/d Rosuvastatin: 2.5mg/d |
| Tani 2005 | Japan | RCT | IVUS | 75 | coronary artery disease | pravastatin: 63±10 Control Group: 62±13 | 6 months | pravastatin:5/10/20 mg/d Control Group: NR |
| Tani 2010 | Japan | Cohort study | IVUS | 84 | Coronary artery disease | 63±10 | 6 months | Pravastatin: 12.5±3.2mg/d |
| Thondapu 2019 | China | RCT | OCT+IVUS | 24 | coronary artery disease | 57.5 | 6/12 months | Rosuvastatin: 10mg/d |
| Tsujita 2015 | Japan | RCT | IVUS | 202 | coronary artery disease | Atorvastatin + ezetimibe: 66±10; Atorvastatin: 67±10 | 9-12 month | Atorvastatin + ezetimibe: atorvastatin 20mg+ ezetimibe 10mg Atorvastatin: 20mg |
| Ueda 2017 | Japan | RCT | IVUS | 108 | Coronary artery disease | 69±10 | 1/3/9 months | Statin monotherapy: atorvastatin 10–20 mg/d； Combination therapy: atorvastatin 10–20 mg and ezetimibe 10 mg/d |
| Wang 2009 | China | CCT | IVUS | 40 | Coronary atherosclerotic plaque +type2 diabetes | Stain group: 65.5±7.4 Control group: 63.3±6.0 | 12 months | Atorvastatin 10~20mg/d or Simvastatin 40~80mg/d or Fluvastatin 40~80mg/d Control Group: NR |
| Xu 2021 | China | RCT | OCT+IVUS | 50 | Coronary artery disease | Atorvastatin: 55.0 ± 10.5 Rosuvastatin: 57.3 ± 8.4 | 6/12 months | atorvastatin: 60mg/d rosuvastatin: 10mg/d |
| Yano 2019 | Japan | Cohort study | OCT | 58 | Acute coronary syndrome | Rosuvastatin: 65.2±6.2 Rosuvastatin + evolocumab: 64.6±5.3 | 3 months | Rosuvastatin: 5mg/d Rosuvastatin + evolocumab: Rosuvastatin 5mg/d + evolocumab 140mg every 2 weeks |
| Yokoyama 2005 | Japan | RCT | IVUS-RF | 42 | Sable angina | Atorvastatin: 62.1±10.2 Control Group:64.4±8 .7 | 6 months | Atorvastatin: 10mg/d Control Group: NR |
| Zhang 2012 | China | RCT | IVUS | 100 | new-onset unstable angina with borderline lesions | Atorvastatin 20mg: 65.5±6.2 Atorvastatin 80mg: 64.5±13.8 | 9 months | Atorvastatin: 20mg/d Atorvastatin: 80mg/d |
| Zhu 2017 | China | Cohort study | OCT | 69 | CHD (coronary heart disease) + lipid rich plaques | low FRS group:52.1±9.7; moderate to high FRS group:59.4±7.6 | 6/12 months | atorvastatin 60 mg/d (AT60), or atorvastatin 20 mg/d (AT20), or rosuvastatin 10 mg/d (RT10) randomly |

# **Supplementary Figures**

**
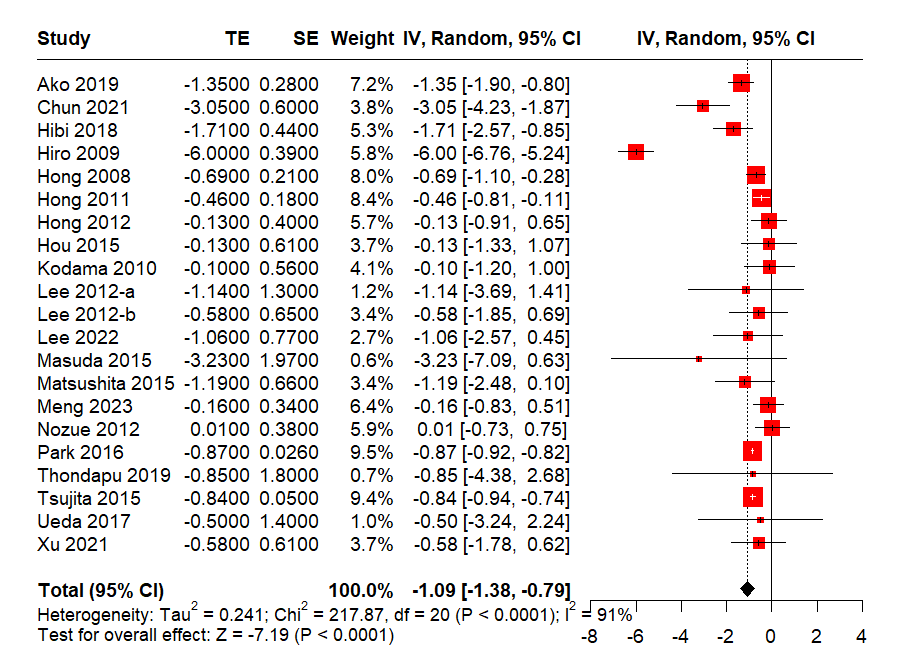
**

**Supplemental Figure 1 Forest plot of mean changes in PAV from baseline**

TE = treatment effect; SE = standard error of treatment effect; IV = inverse variance.


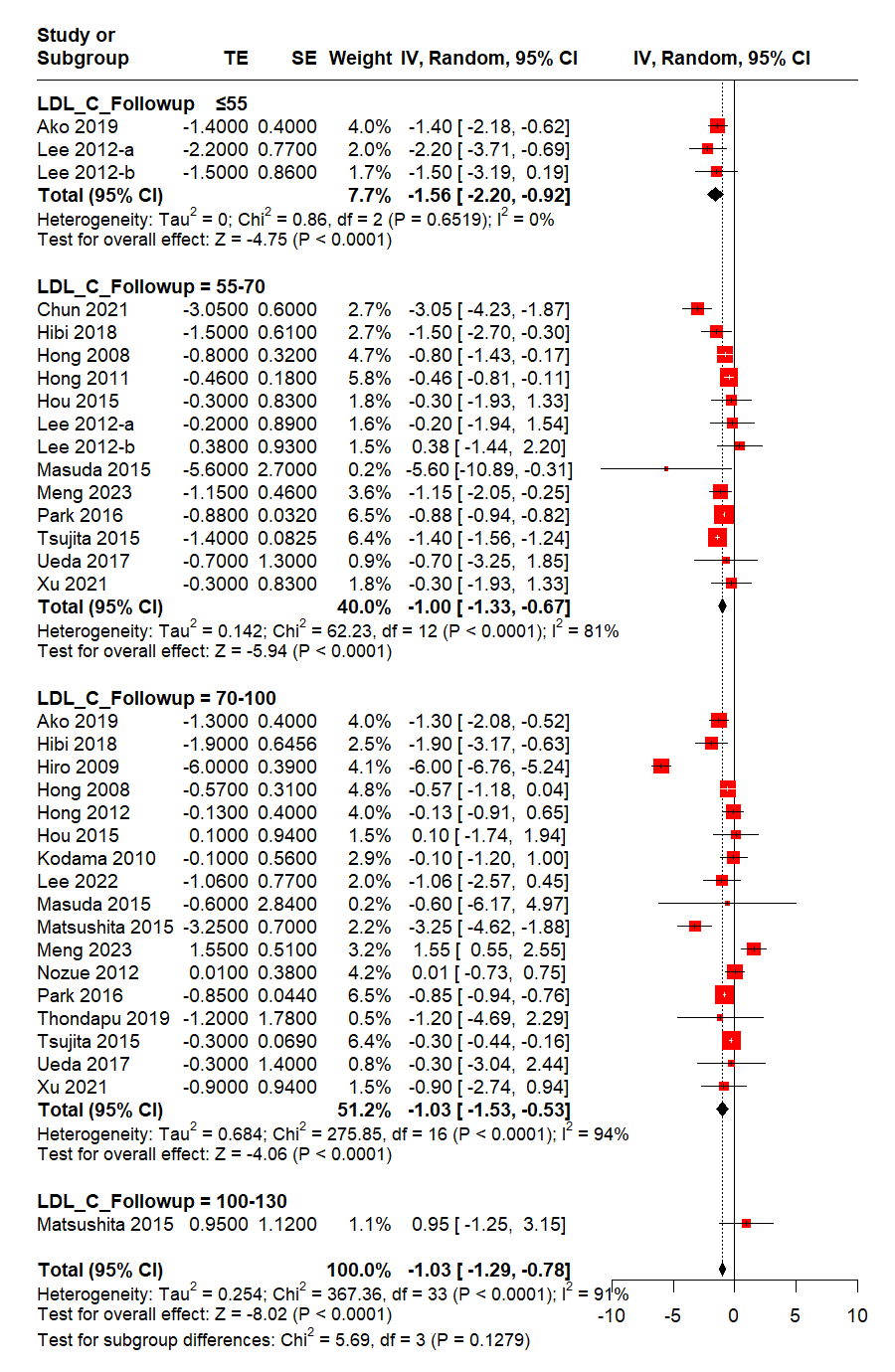


**Supplemental Figure 2 Change in PAV according to different levels of LDL-C at follow-up (Hiro 2009 study retained)**

TE = treatment effect; SE = standard error of treatment effect; IV = inverse variance.


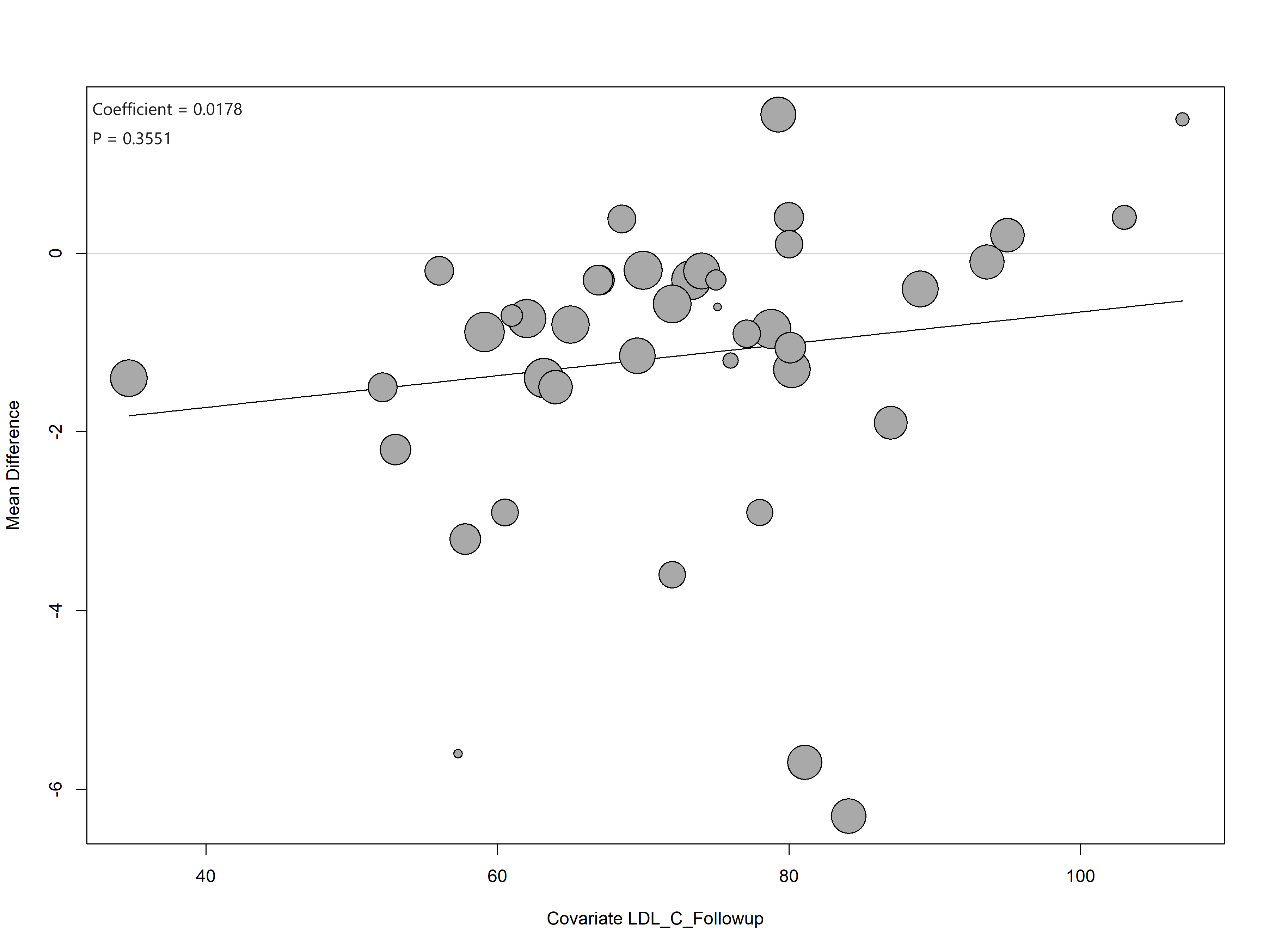


**Supplemental Figure 3 Effect of LDL-C levels at follow-up on PAV (Hiro2009 study retained)**


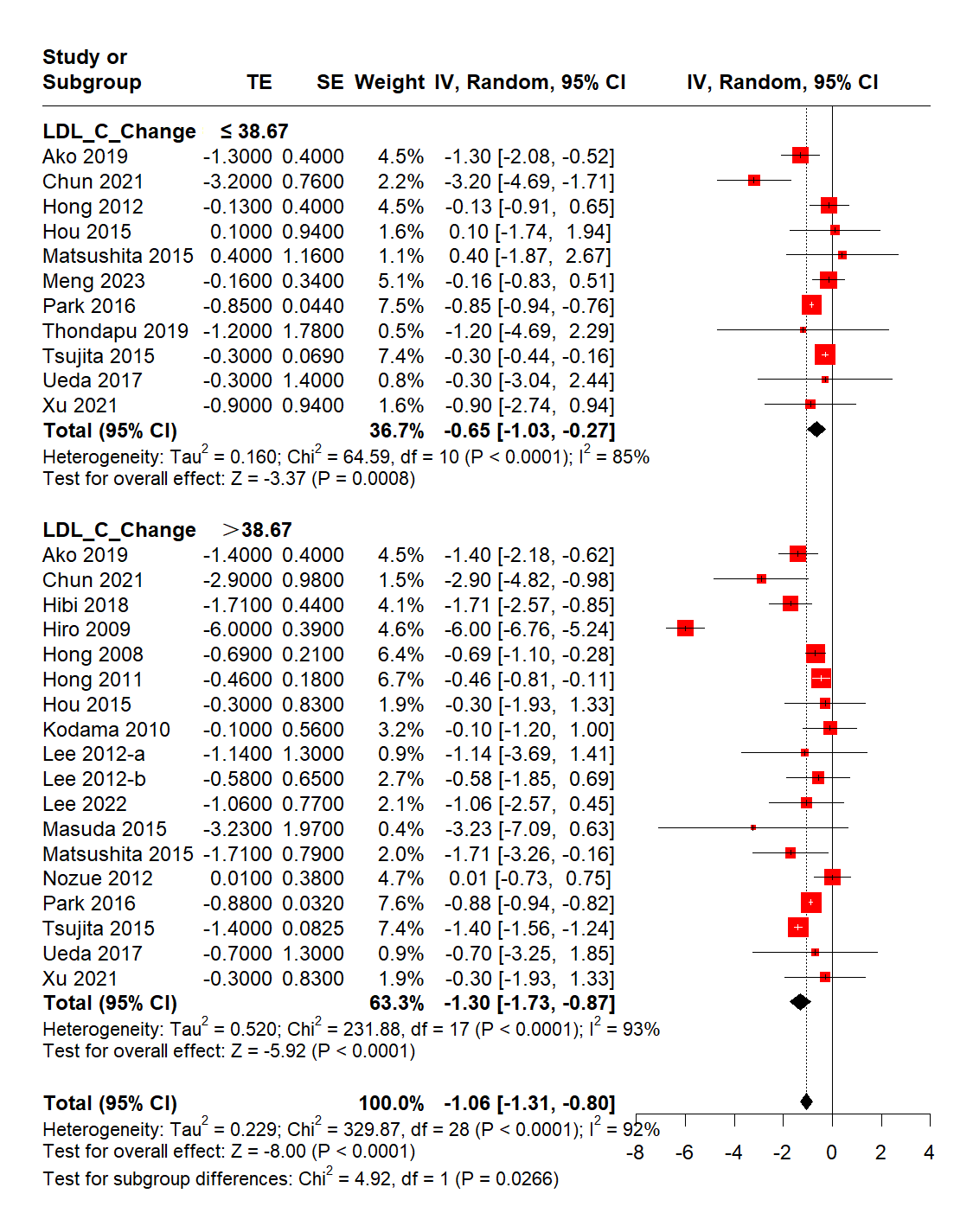


**Supplemental Figure 4 Change in PAV according to different levels of LDL C change values (Hiro 2009 study retained)**

TE = treatment effect; SE = standard error of treatment effect; IV = inverse variance.


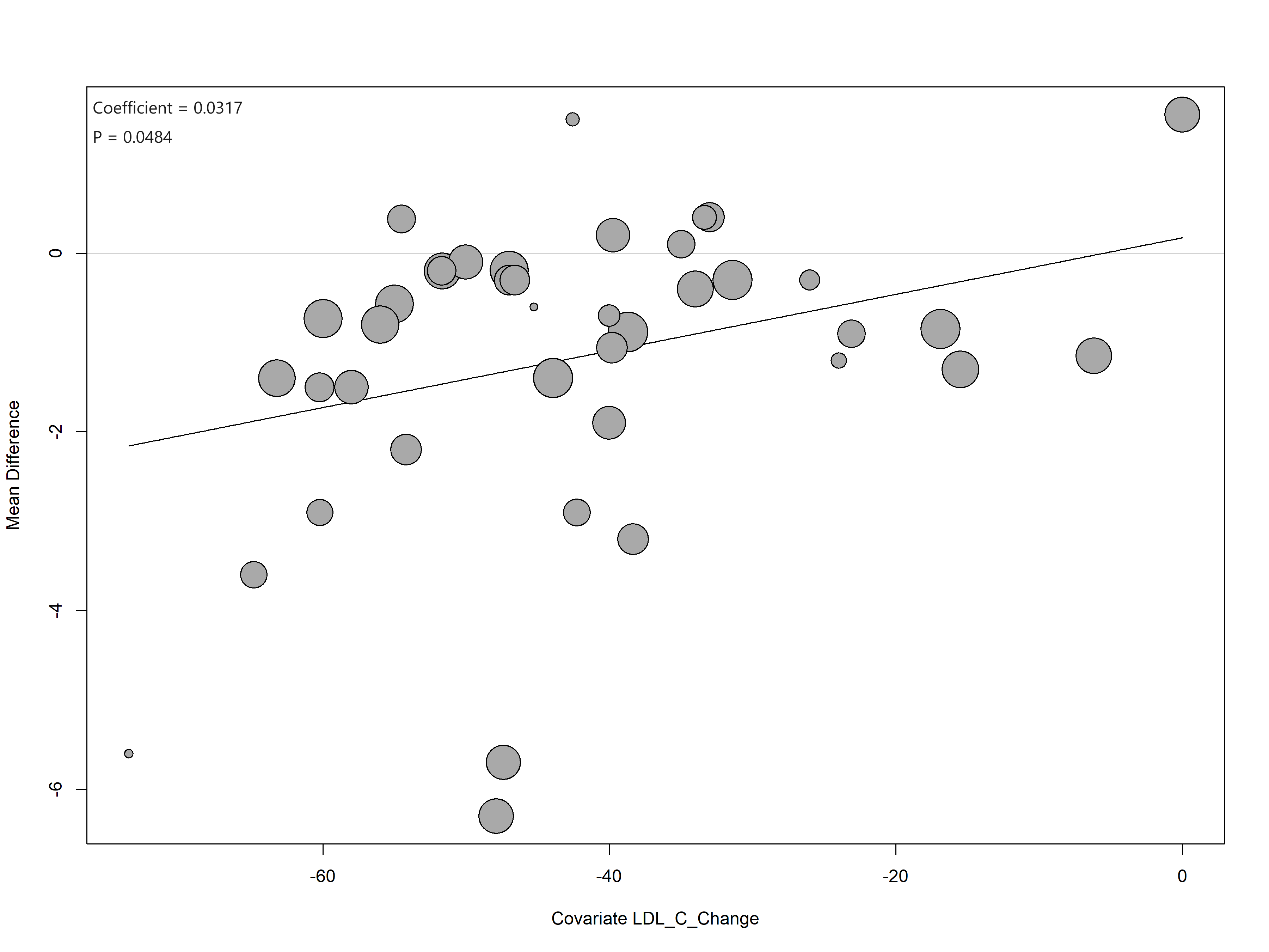


**Supplemental Figure 5 Effect of LDL-C levels change on PAV (Hiro2009 study retained)**

**Supplemental Figure 6 Sensitivity analysis in PAV**


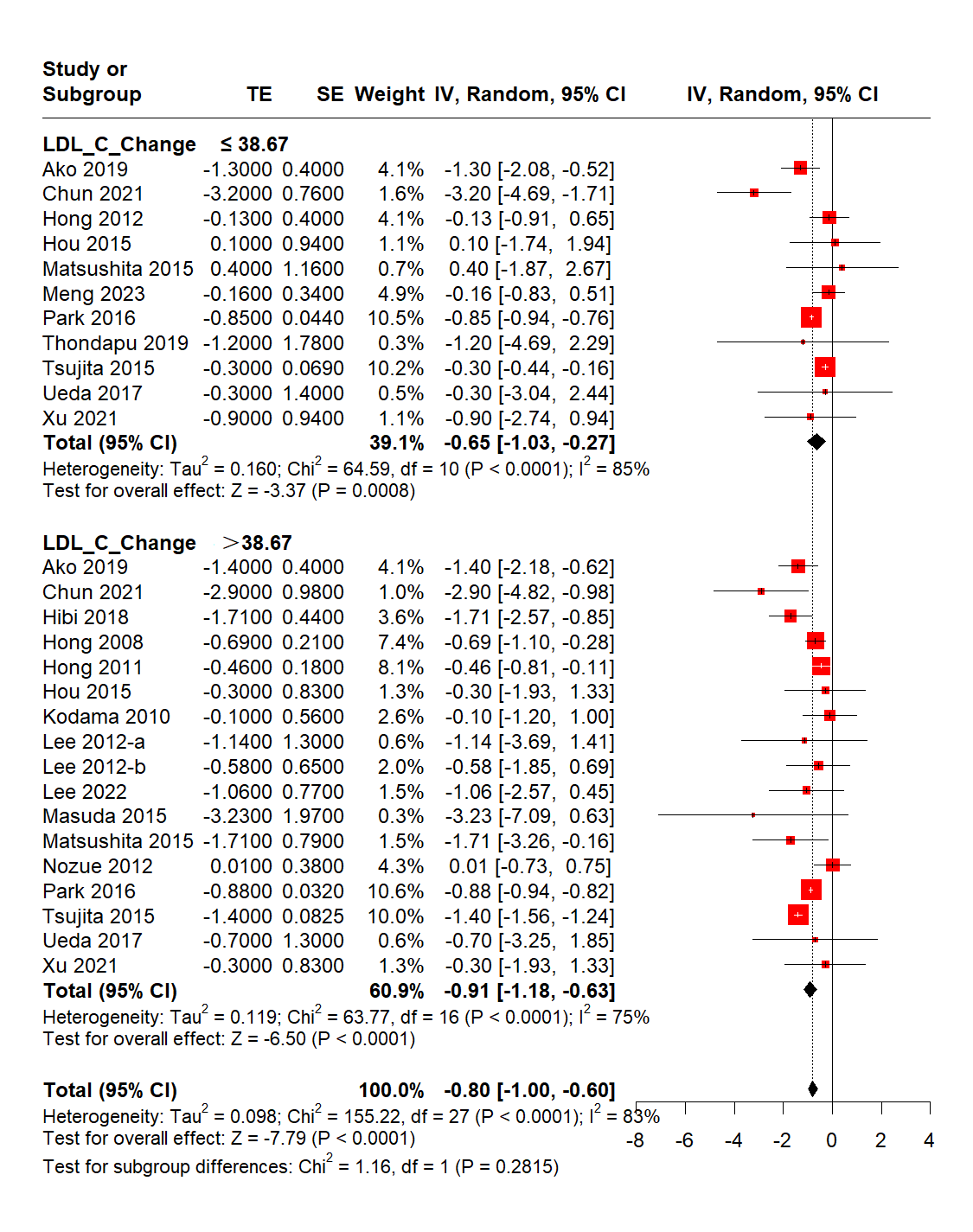


**Supplemental Figure 7 Change in PAV according to different levels of LDL‑C change values (Hiro 2009 study excluded)**

TE = treatment effect; SE = standard error of treatment effect; IV = inverse variance.


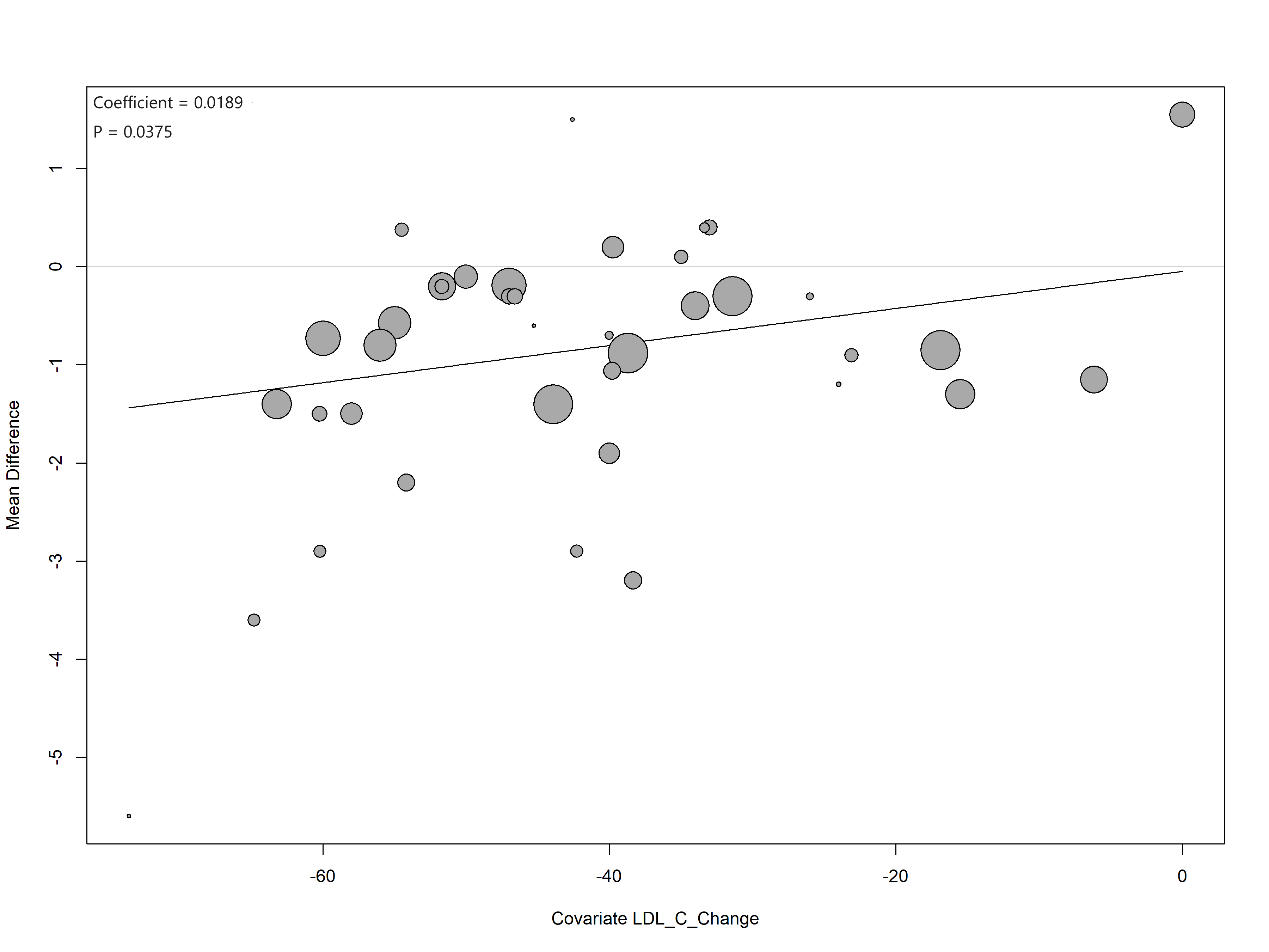


**Supplemental Figure 8 Effect of LDL-C levels change on PAV (Hiro2009 study excluded)**


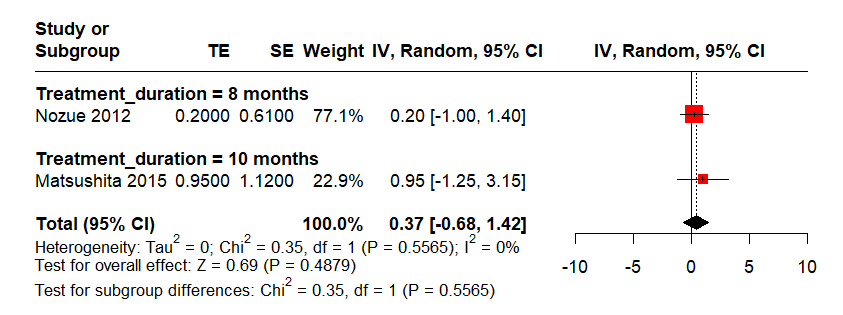


**Supplemental Figure 9 Subgroup analysis of PAV according to different treatment duration stratified by Low-intensity statin therapy**

TE = treatment effect; SE = standard error of treatment effect; IV = inverse variance.


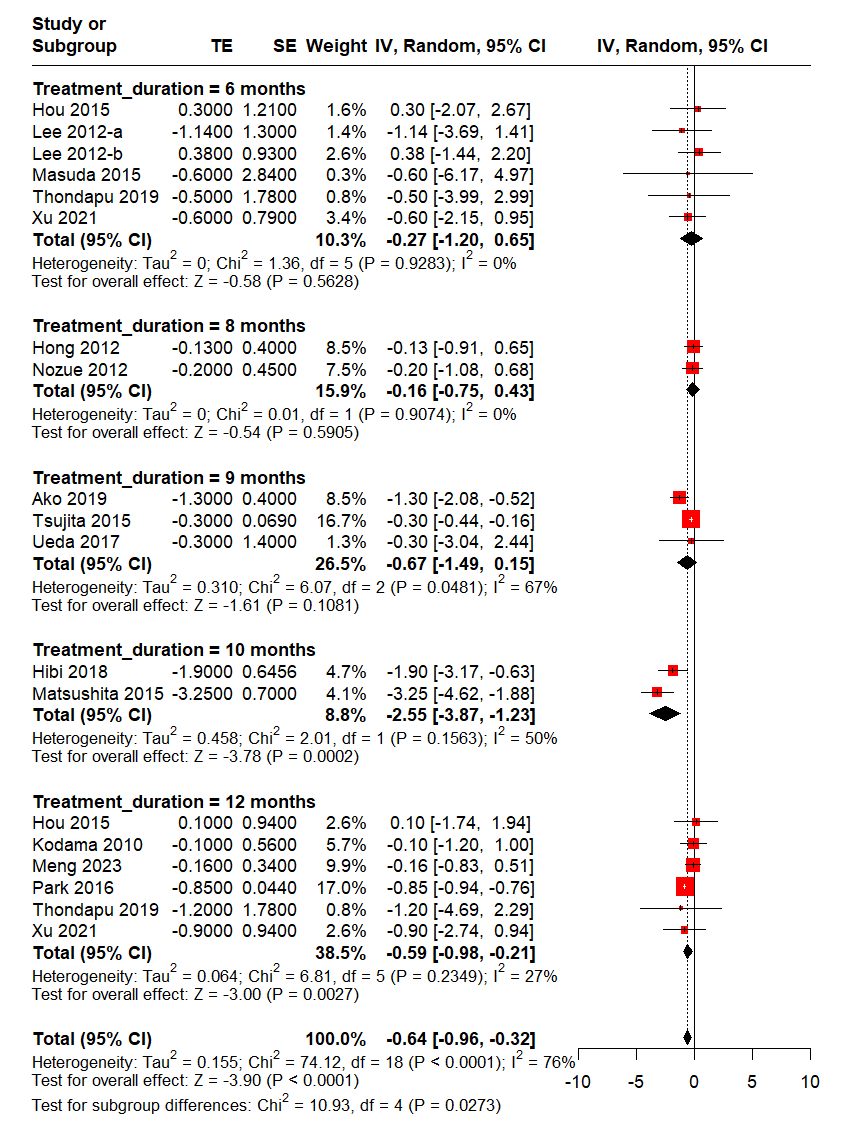


**Supplemental Figure 10 Subgroup analysis of PAV according to different treatment duration stratified by Moderate-intensity statin therapy**

TE = treatment effect; SE = standard error of treatment effect; IV = inverse variance.


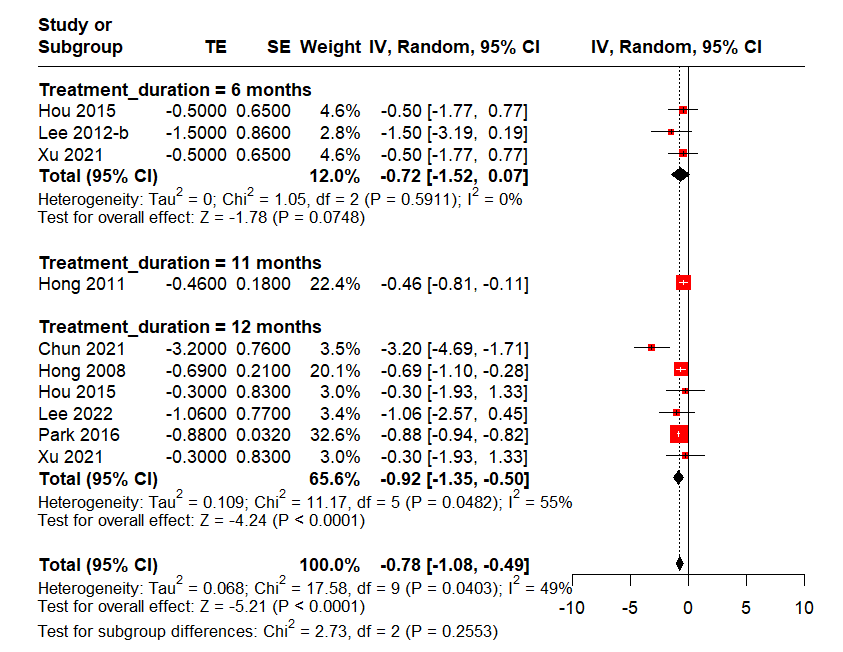


**Supplemental Figure 11 Subgroup analysis of PAV according to different treatment duration stratified by High-intensity statin therapy**

TE = treatment effect; SE = standard error of treatment effect; IV = inverse variance.


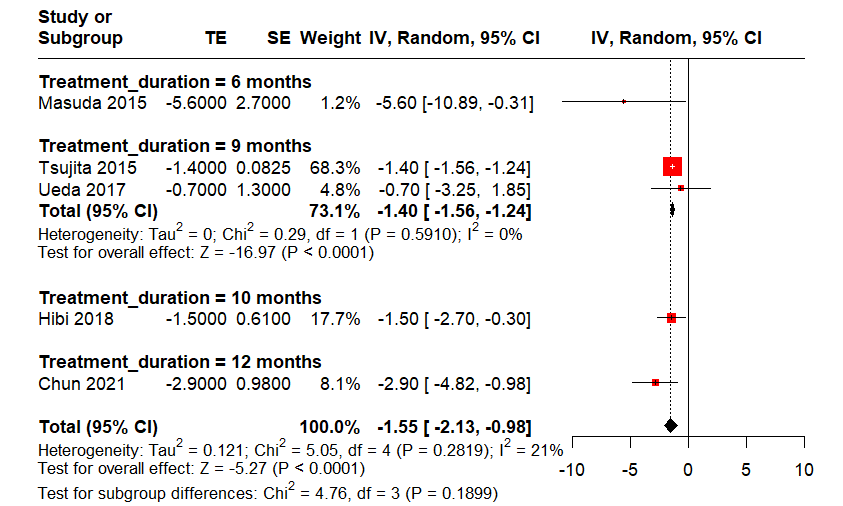


**Supplemental Figure 12 Subgroup analysis of PAV according to different treatment duration stratified by Moderate-intensity statin + ezetimibe therapy**

TE = treatment effect; SE = standard error of treatment effect; IV = inverse variance.


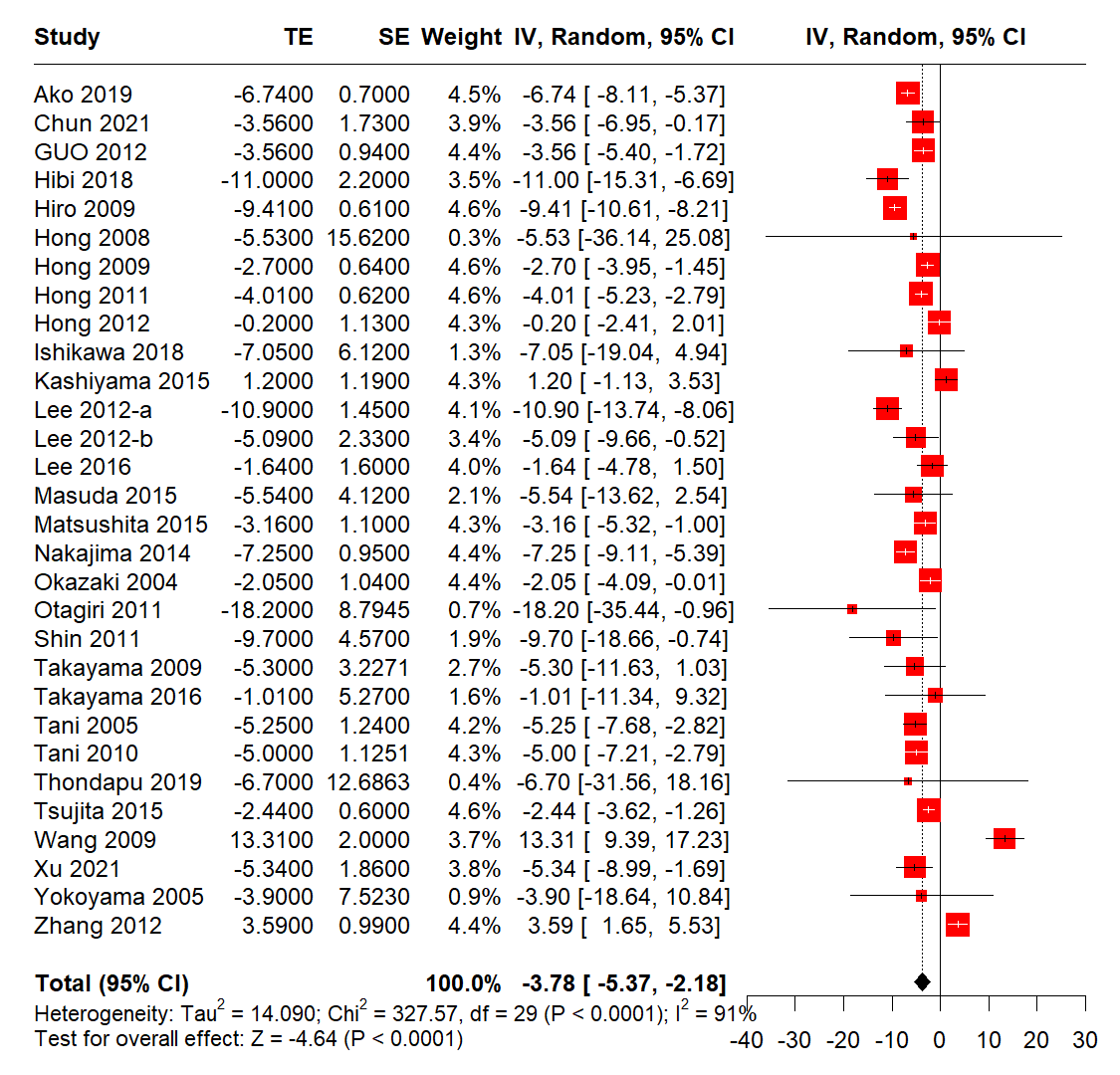


**Supplemental Figure 13 Forest plot of mean changes in TVA from baseline**

TE = treatment effect; SE = standard error of treatment effect; IV = inverse variance.


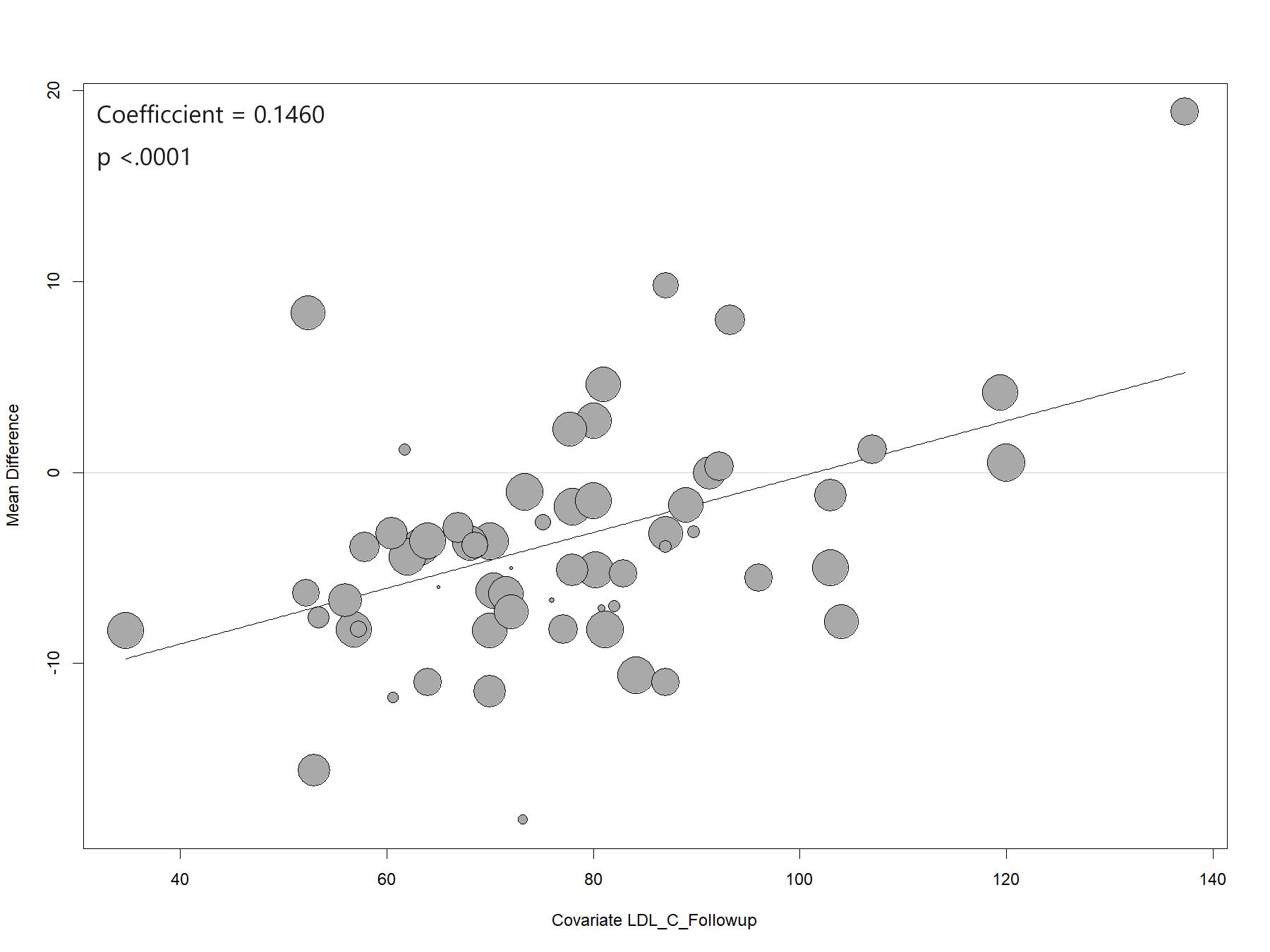


**Supplemental Figure 14 Effect of LDL-C levels at follow-up on TAV**


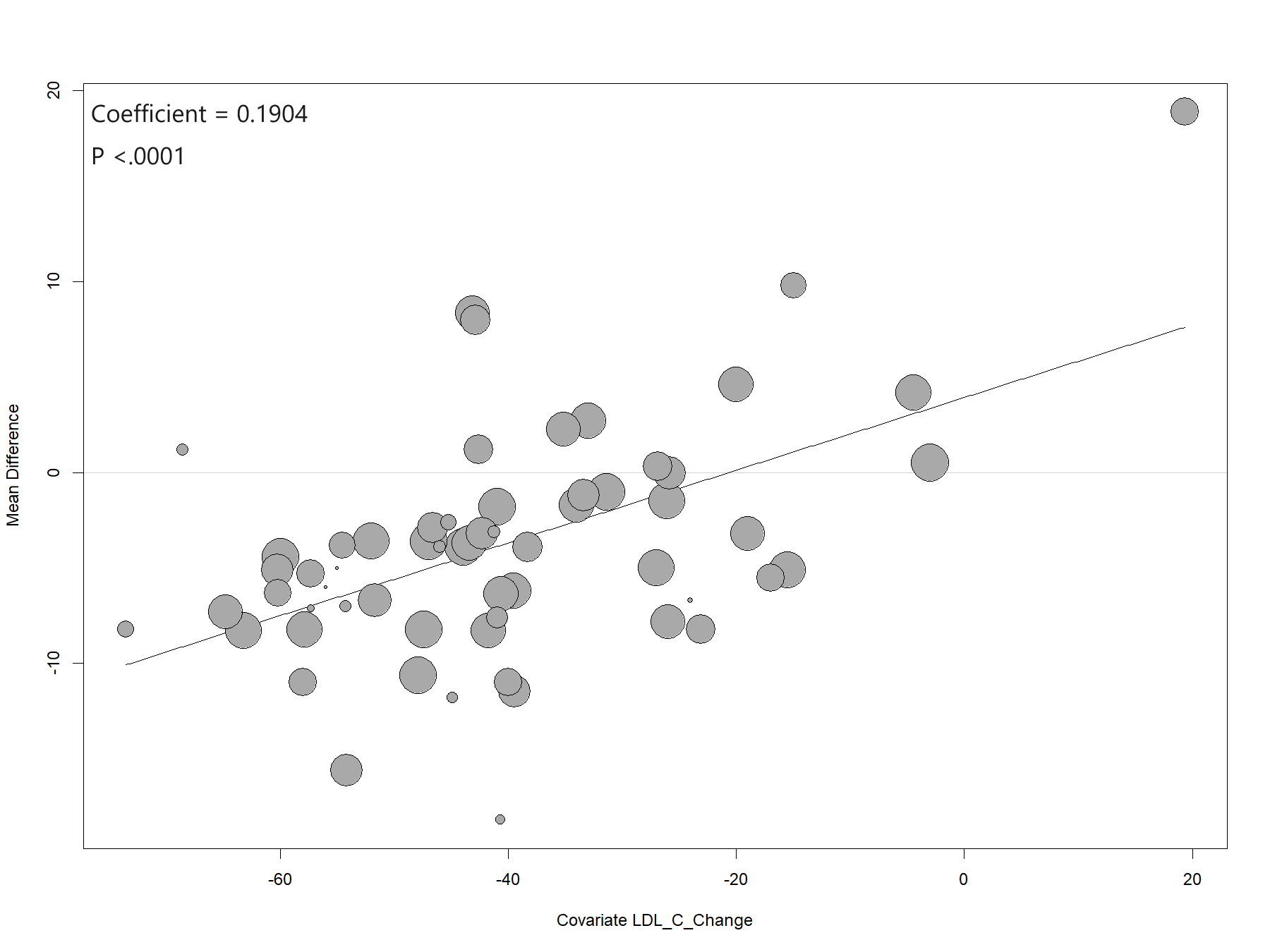


**Supplemental Figure 15 Effect of LDL-C levels change between baseline and follow-up on TAV**


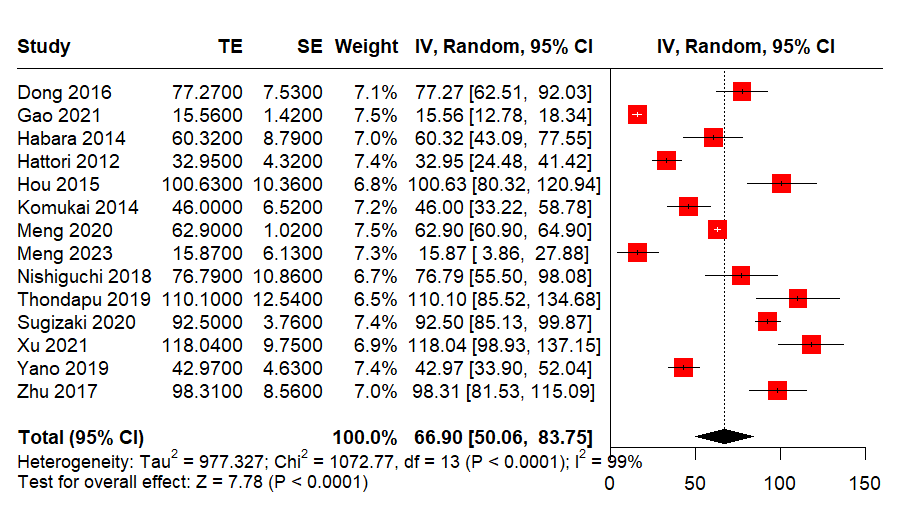


**Supplemental Figure 16 Forest plot of mean changes in FCT from baseline**

TE = treatment effect; SE = standard error of treatment effect; IV = inverse variance.


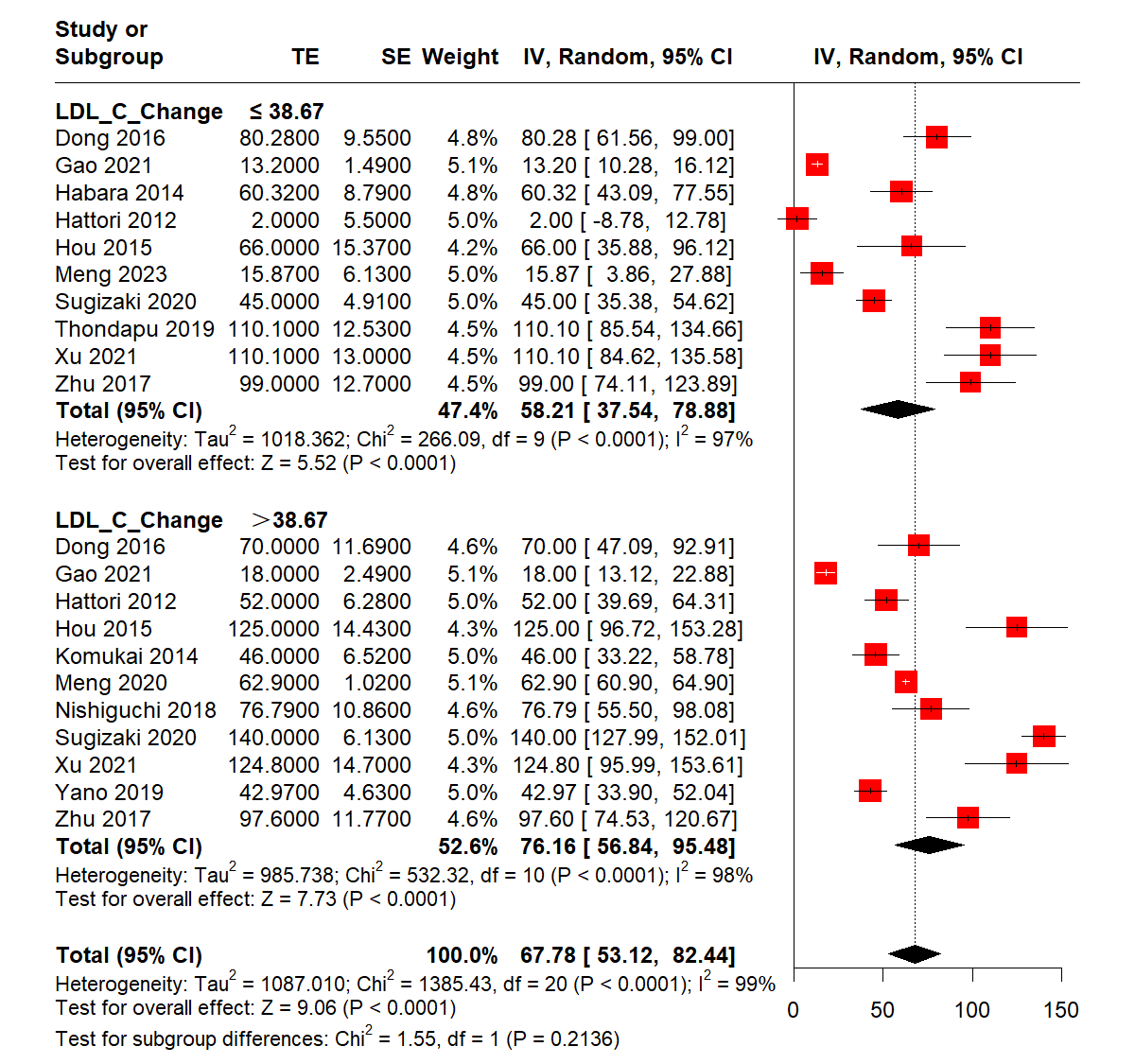


**Supplemental Figure 17 Subgroup analysis of change in FCT according to different levels of LDL-C change value**

TE = treatment effect; SE = standard error of treatment effect; IV = inverse variance.


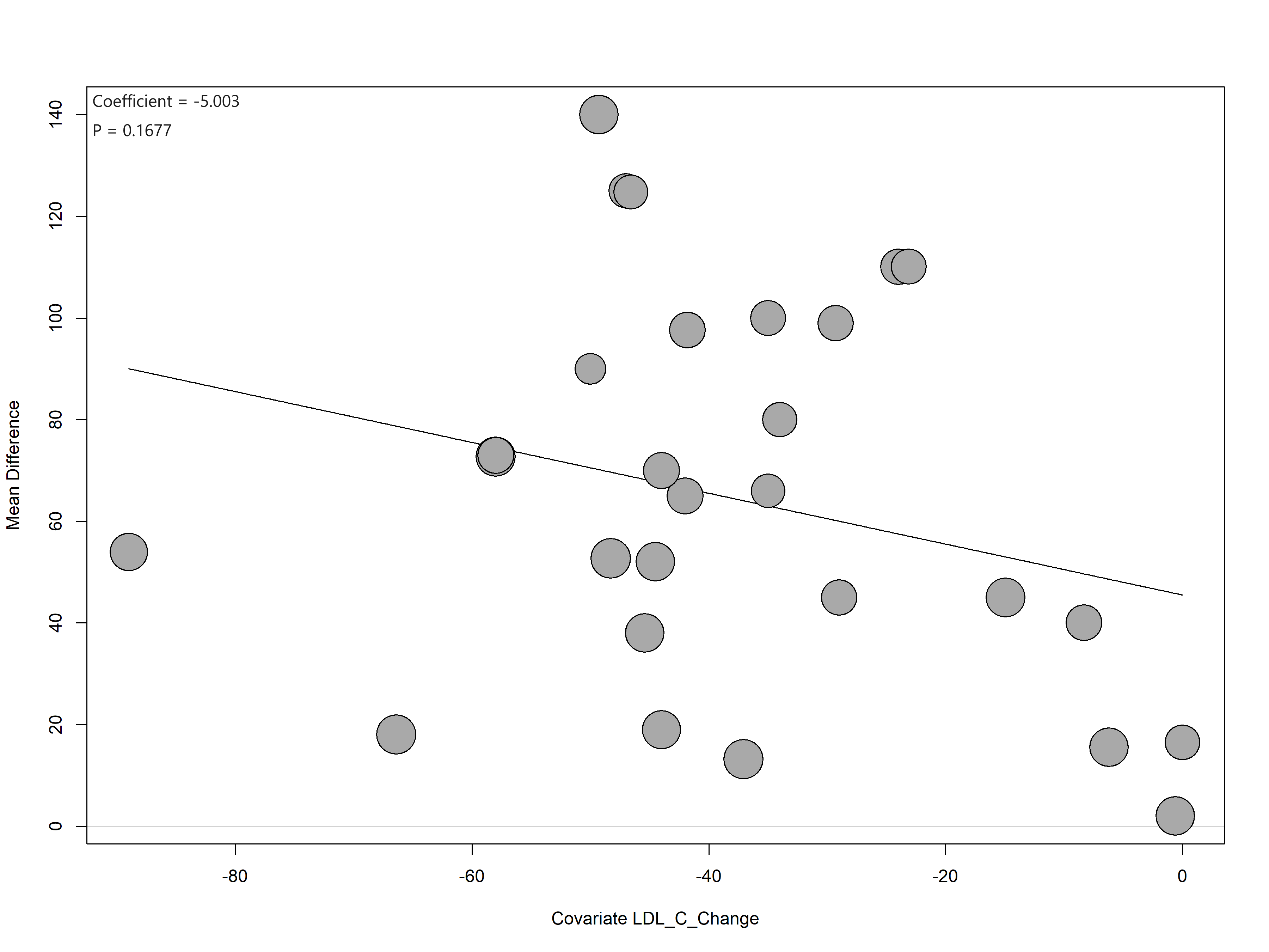


**Supplemental Figure 18 Effect of LDL-C levels change on FCT**


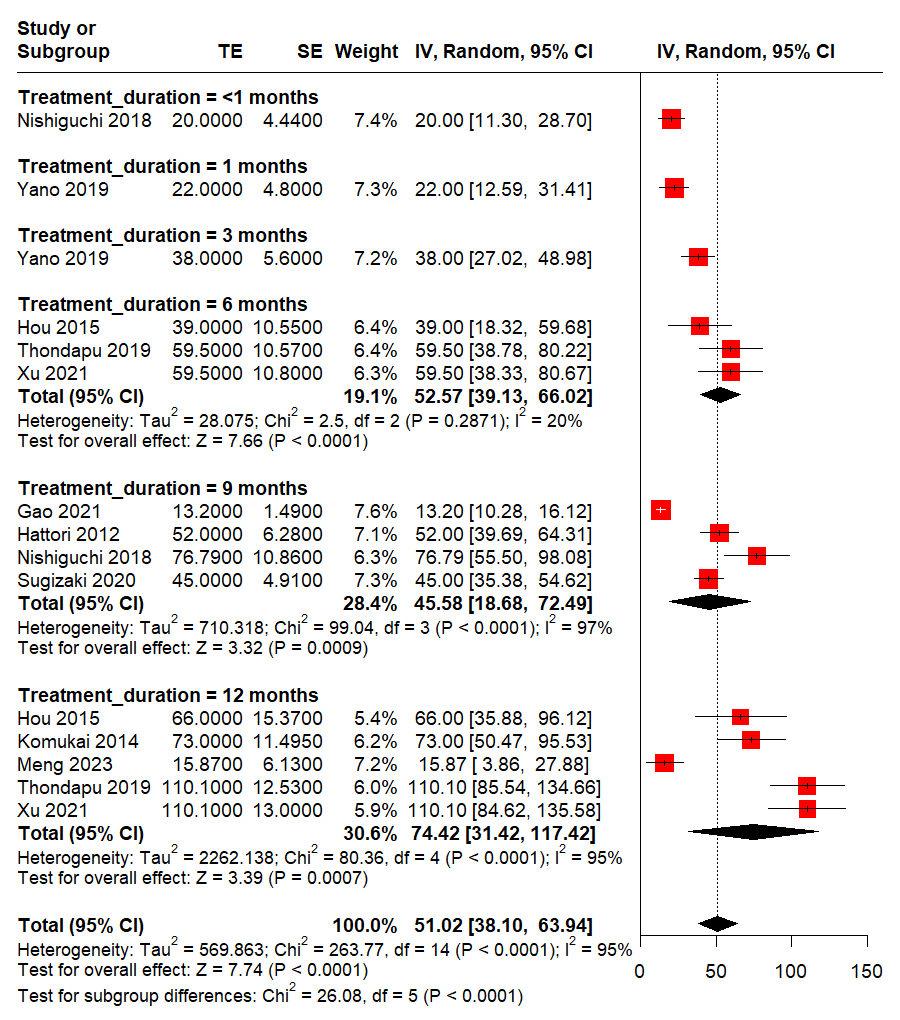


**Supplemental Figure 19 Subgroup analysis of FCT according to different treatment duration stratified by Moderate-intensity statin therapy**

TE = treatment effect; SE = standard error of treatment effect; IV = inverse variance.


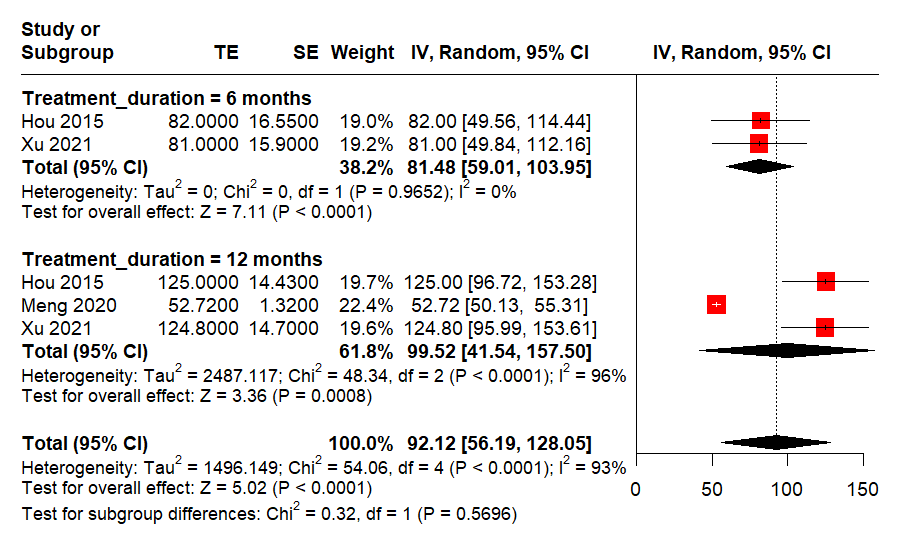


**Supplemental Figure 20 Subgroup analysis of FCT according to different treatment duration stratified by High-intensity statin therapy**

TE = treatment effect; SE = standard error of treatment effect; IV = inverse variance.


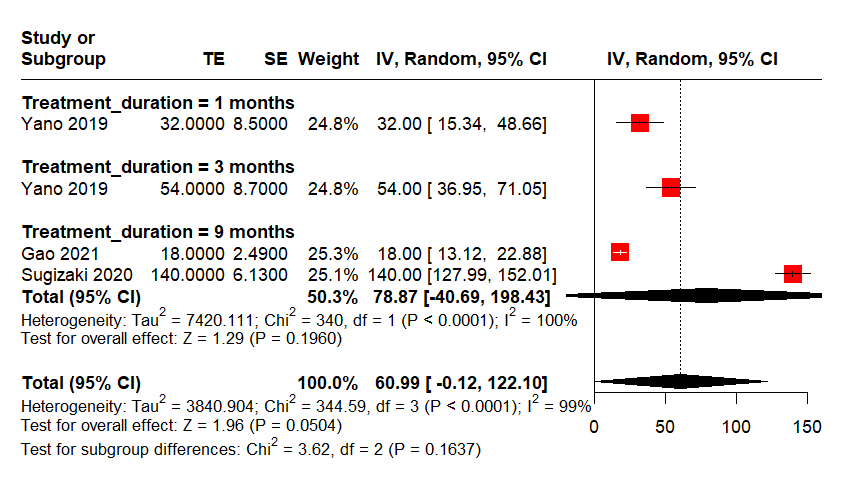


**Supplemental Figure 21 Subgroup analysis of FCT according to different treatment duration stratified by Moderate-intensity statin + PCSK9i therapy**

TE = treatment effect; SE = standard error of treatment effect; IV = inverse variance.


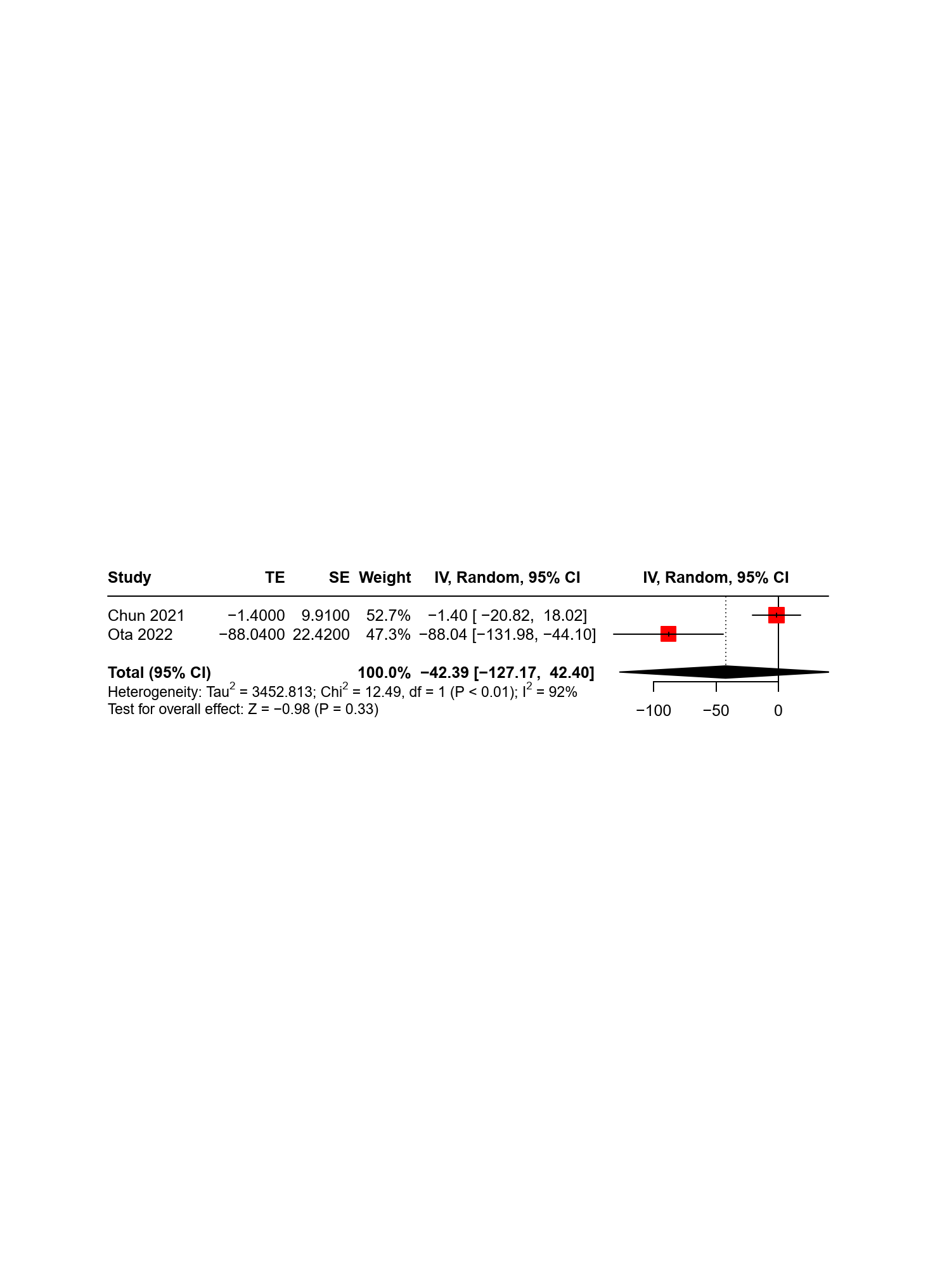


**Supplemental Figure 22 Forest plot of mean changes in LCBI from baseline**

TE = treatment effect; SE = standard error of treatment effect; IV = inverse variance.

**Supplemental Figure 23 Sensitivity analysis in TAV**

**Supplemental Figure 24 Sensitivity analysis in FCT**


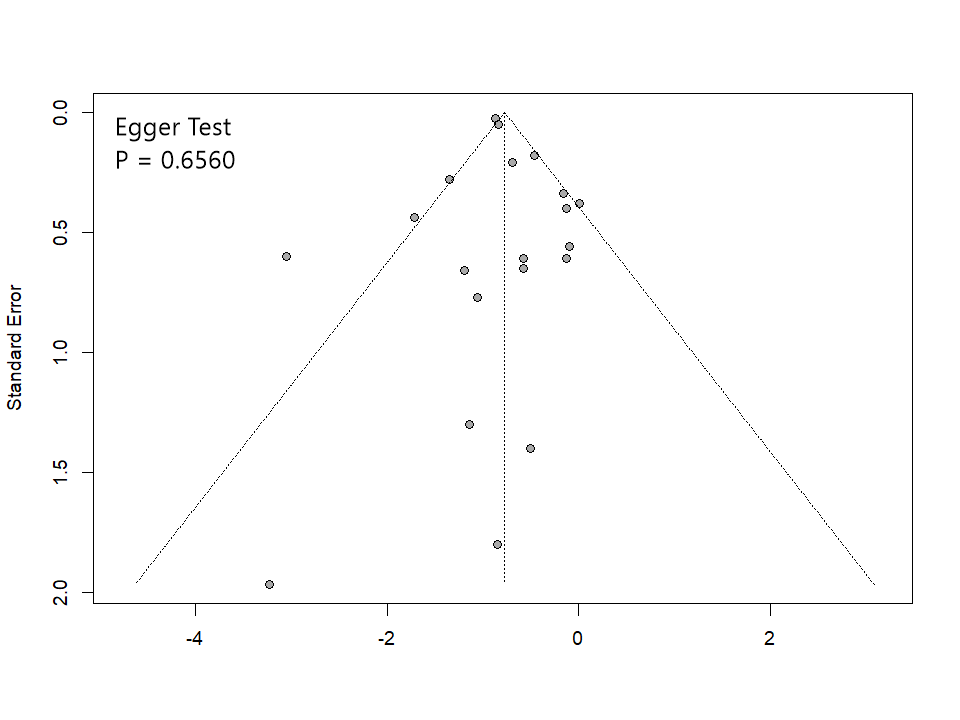


**Supplemental Figure 25 Funnel plot in PAV**


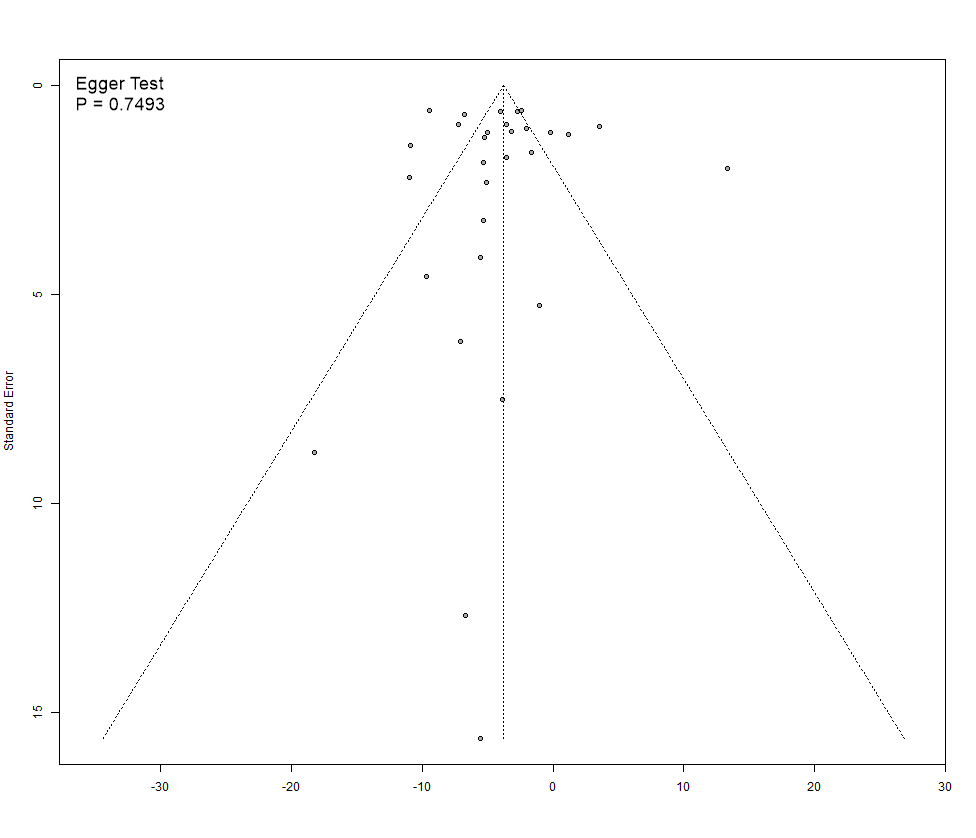


**Supplemental Figure 26 Funnel plot in TAV**


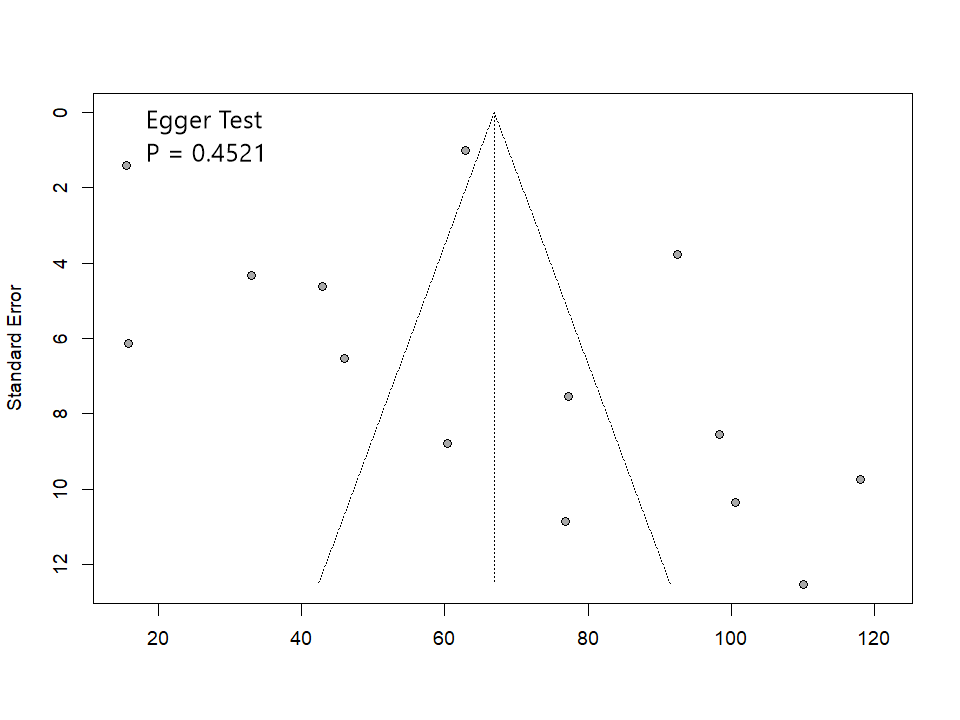


**Supplemental Figure 27 Funnel plot in FCT**

# **Supplementary Tables**

**Supplemental Table 1 Results of regression analysis between LDL-C levels and change percent in PAV (Hiro2009 study retained) (Number of studies:21)**

| **Variable** | **Coefficient** | **R^2^** | **P value** |
| --- | --- | --- | --- |
| Follow-up value | 0.0178 | 0.00% | 0.3551 |
| Change value | 0.0317 | 5.87% | 0.0484 |

Follow-up value：y = -2.4382+ 0.0178x

Change value：y = 0.1729 + 0.0317x

**Supplemental Table 2 Results of regression analysis between LDL-C levels and change percent in PAV (Hiro2009 study excluded) (Number of studies:20)**

| **Variable** | **Coefficient** | **R^2^** | **P value** |
| --- | --- | --- | --- |
| Follow-up value | 0.0296 | 28.20% | 0.0044 |
| Change value | 0.0189 | 3.96% | 0.0375 |

Follow-up value：y = -2.9066 + 0.0296x

Change value：y = -0.0468 + 0.0189x

**Supplemental Table 3 Results of regression analysis between other covariates and change percent in PAV**

| **Variable** | **No. of studies** | **Coefficient** | **P value** |
| --- | --- | --- | --- |
| **Patients** | 15 | - | - |
| ACS (reference) | - | - | - |
| CAD | - | -0.0276 | 0.9532 |
| **Gender** | 20 | -2.8398 | 0.0778 |
| **Age** | 20 | 0.0034 | 0.7852 |
| **Smoker** | 19 | -0.9457 | 0.4979 |
| **Hypertension** | 20 | 1.5425 | 0.0536 |
| **Diabetes** | 20 | 0.9432 | 0.1806 |
| **HbA1C** | 5 | - | - |
| Follow-up value | - | -3.0119 | 0.1175 |
| Change value | - | -0.1627 | 0.6789 |
| **HDL-C** | 20 | - | - |
| Follow-up value | - | -0.0054 | 0.8966 |
| Change value | - | -0.0065 | 0.8156 |
| **LDL-C baseline** | 20 | 0.0001 | 0.9897 |
| **Treatment duration** | 20 | - | - |
| 6 months(reference) | - | - | - |
| 8 months | - | 0.6669 | 0.2020 |
| 9 months | - | -0.2592 | 0.5656 |
| 10 months | - | -1.0095 | 0.0696 |
| 11 months | - | 0.2852 | 0.6061 |
| 12 months | - | 0.0160 | 0.9679 |
| **Publication years** | 20 | -0.0463 | 0.1952 |
| **Country** | 20 | - | - |
| Japan (reference) | - | - | - |
| China | - | 1.7915 | 0.0732 |
| South Korea | - | 0.3303 | 0.7412 |
| **Study design** | 20 | - | - |
| Trial (reference) | - | - | - |
| Observational | - | 0.5979 | 0.1164 |

**Supplemental Table 4 Results of regression analysis between LDL-C and changes in FCT (Number of studies:14)**

| **Variable** | **Coefficient** | **R^2^** | **P value** |
| --- | --- | --- | --- |
| Follow-up value | -0.8128 | 13.64% | 0.0283 |
| Change value | -0.5003 | 3.22% | 0.1677 |

Follow-up value：y = 122.4290 - 0.8128x

Change value：y = 45.5045 - 0.5003x

**Supplemental Table 5 Results of regression analysis between other factor and changes in FCT**

| **Variable** | **No. of studies** | **Coefficient** | **P value** |
| --- | --- | --- | --- |
| **Patients** | 13 | - | - |
| ACS (reference) | - | - | - |
| CAD | - | 31.9816 | 0.0215 |
| **Gender** | 13 | -150.8901 | 0.0148 |
| **Age** | 13 | -4.2255 | <.0001 |
| **Smoker** | 13 | 68.2788 | 0.2076 |
| **Hypertension** | 13 | -74.0180 | 0.3367 |
| **Diabetes** | 13 | 23.1515 | 0.4087 |
| **HbA1C** | 5 | - | - |
| Follow-up value | - | 4.8118 | 0.2835 |
| Change value | - | -7.2111 | 0.8950 |
| **HDL-C** | 13 | - | - |
| Follow-up value |  | -2.3104 | 0.0828 |
| Change value |  | -3.2027 | 0.0344 |
| **LDL-C baseline** | 14 | -0.2998 | 0.5437 |
| **FCT baseline** | 14 | -0.7119 | <.0001 |
| **Treatment duration** | 14 | - | - |
| <1 months (reference) | - | - | - |
| 1 months | - | 6.8924 | 0.8667 |
| 3 months | - | 25.8457 | 0.5295 |
| 6 months | - | 42.0102 | 0.2428 |
| 9 months | - | 33.0464 | 0.3468 |
| 12 months | - | 53.6710 | 0.1200 |
| **Publication years** | 14 | 0.0017 | 0.9994 |
| **Country** | 14 | - | - |
| Japan (reference) | - | - | - |
| China | - | 12.2893 | 0.4080 |
| **Study design** | 14 | - | - |
| Trial (reference) | - | - | - |
| Observational | - | -5.0910 | 0.7371 |

**Supplemental Table 6 Subgroup analysis of changes in FCT according to other factors**

| **Subgroup factors** | **No. of studies** | **Mean** | **95% CI** | **I^2** | **P value** |
| --- | --- | --- | --- | --- | --- |
| **Patients** | 13 | - | - | - | - |
| ACS | - | 48.24 | [30.12, 66.35] | 95% | 0.012 |
| CAD | - | 85.61 | [62.67, 108.56] | 96% |  |
| **Gender** | 13 | - | - | - | - |
| 40%-70% Male | - | 82.97 | [44.23, 121.72] | 97% | 0.178 |
| 70%-100% Male | - | 53.01 | [33.11, 72.91] | 99% |  |
| **Age** | 13 | - | - | - | - |
| 50-60 years | - | 99.79 | [84.87, 114.72] | 69% | <0.0001 |
| 60-70 years | - | 43.66 | [23.00, 64.32] | 99% |  |
| **HDL-C Change** | 13 | - | - | - | - |
| Decreased or stayed unchanged | - | 78.92 | [31.05, 126.79] | 98% | 0.319 |
| Increased | - | 52.51 | [32.36, 72.66] | 99% |  |
| **FCT baseline** | 14 |  |  |  |  |
| ＜65μm | - | 94.79 | [76.29, 113.29] | 95% | <0.0001 |
| ＞65μm | - | 40.41 | [26.61, 54.21] | 94% |  |

**Supplemental Table 7 Effect of LDL-C on FCT changes under patient subgroups**

| **Subgroup factors** | **Mean** | **95% CI** | **I^2** | **P value** |
| --- | --- | --- | --- | --- |
| **ACS** | - | - | - | - |
| LDL-C Follow-up value | - | - | - | - |
| ≤55 | 65.31 | [47.38, 83.24] | 78% | 0.058 |
| 55-70 | 53.83 | [28.13, 79.53] | 94% |  |
| 70-100 | 34.30 | [16.02, 52.58] | 79% |  |
| LDL-C Change value | - | - | - | - |
| ≤1mmol/L | 15.87 | [3.86, 27.88] | - | ＜0.0001 |
| ＞1mmol/L | 55.79 | [42.34, 69.24] | 88% |  |
| **CAD** | - | - | - | - |
| LDL-C Follow-up value | - | - | - | - |
| ≤55 | 140.00 | [127.99, 152.01] | - | ＜0.0001 |
| 55-70 | 115.78 | [99.13, 132.44] | 7% |  |
| 70-100 | 76.19 | [57.60, 94.78] | 89% |  |
| 100-130 | 19.58 | [-17.56, 56.71] | 88% |  |
| LDL-C Change value | - | - | - | - |
| ≤1mmol/L | 70.58 | [42.74, 98.42] | 95% | 0.189 |
| ＞1mmol/L | 101.21 | [64.96, 137.46] | 96% |  |

**Supplemental Table 8 Effect of LDL-C on FCT changes under Gender subgroups**

| **Subgroup factors** | **Mean** | **95% CI** | **I^2** | **P value** |
| --- | --- | --- | --- | --- |
| **40%-70% Male** | - | - | - | - |
| LDL-C Follow-up value | - | - | - | - |
| ≤55 | 18.00 | [13.12, 22.88] | - | ＜0.0001 |
| 55-70 | 124.90 | [104.72, 145.08] | 0% |  |
| 70-100 | 86.88 | [66.78, 106.98] | 76% |  |
| LDL-C Change value | - | - | - | - |
| ≤1mmol/L | 85.30 | [63.46, 107.14] | 76% | 0.955 |
| ＞1mmol/L | 83.45 | [23.32, 143.59] | 97% |  |
| **70%-100% Male** | - | - | - | - |
| LDL-C Follow-up value | - | - | - | - |
| ≤55 | 65.31 | [47.38, 83.24] | 78% | 0.080 |
| 55-70 | 62.70 | [38.33, 87.08] | 93% |  |
| 70-100 | 42.00 | [22.59, 61.40] | 95% |  |
| 100-130 | 19.58 | [-17.56, 56.71] | 88% |  |
| LDL-C Change value | - | - | - | - |
| ≤1mmol/L | 29.60 | [11.54, 47.65] | 92% | 0.005 |
| ＞1mmol/L | 59.99 | [48.55, 71.42] | 87% |  |

**Supplemental Table 9 Effect of LDL-C on FCT changes under Age subgroups**

| **Subgroup factors** | **Mean** | **95% CI** | **I^2** | **P value** |
| --- | --- | --- | --- | --- |
| **50-60 years** | - | - | - | - |
| LDL-C Follow-up value | - | - | - | - |
| 55-70 | 115.78 | [99.13, 132.44] | 7% | 0.056 |
| 70-100 | 88.37 | [65.73, 111.01] | 82% |  |
| LDL-C Change value | - | - | - | - |
| ≤1mmol/L | 93.37 | [77.33, 109.42] | 54% | 0.527 |
| ＞1mmol/L | 103.26 | [77.18, 129.34] | 76% |  |
| **60-70 years** | - | - | - | - |
| LDL-C Follow-up value | - | - | - | - |
| ≤55 | 48.16 | [5.92, 90.41] | 99% | 0.494 |
| 55-70 | 53.83 | [28.13, 79.53] | 94% |  |
| 70-100 | 38.93 | [21.54, 56.31] | 93% |  |
| 100-130 | 19.58 | [-17.56, 56.71] | 88% |  |
| LDL-C Change value | - | - | - | - |
| ≤1mmol/L | 21.13 | [5.28, 36.98] | 91% | 0.036 |
| ＞1mmol/L | 49.15 | [28.32, 69.97] | 98% |  |

**Supplemental Table 10 Effect of LDL-C on FCT changes under HDL-C Change subgroups**

| **Subgroup factors** | **Mean** | **95% CI** | **I^2** | **P value** |
| --- | --- | --- | --- | --- |
| **Decreased or stayed unchanged** | - | - | - | - |
| LDL-C Follow-up value | - | - | - | - |
| 55-70 | 115.78 | [99.13, 132.44] | 7% | ＜0.0001 |
| 70-100 | 83.15 | [57.71, 108.59] | 83% |  |
| 100-130 | 2.00 | [-8.78, 12.78] | - |  |
| LDL-C Change value |  |  |  |  |
| ≤1mmol/L | 70.92 | [22.11, 119.73] | 97% | 0.252 |
| ＞1mmol/L | 103.26 | [77.18, 129.34] | 76% |  |
| **Increased** | - | - | - | - |
| LDL-C Follow-up value | - | - | - | - |
| ≤55 | 48.16 | [5.92, 90.41] | 99% | 0.889 |
| 55-70 | 53.83 | [28.13, 79.53] | 94% |  |
| 70-100 | 47.62 | [27.79, 67.45] | 95% |  |
| 100-130 | 40.00 | [16.93, 63.07] | - |  |
| LDL-C Change value | - | - | - | - |
| ≤1mmol/L | 47.77 | [16.39, 79.15] | 96% | 0.943 |
| ＞1mmol/L | 49.15 | [28.32, 69.97] | 98% |  |

**Supplemental Table 11 Effect of LDL-C on FCT changes under FCT baseline subgroups**

| **Subgroup factors** | **Mean** | **95% CI** | **I^2** | **P value** |
| --- | --- | --- | --- | --- |
| **FCT Baseline < 65μm** | - | - | - | - |
| LDL-C Follow-up value | - | - | - | - |
| ≤55 | 106.09 | [40.15, 172.04] | 99% | 0.712 |
| 55-70 | 99.37 | [54.60, 144.13] | 95% |  |
| 70-100 | 82.72 | [56.21, 109.22] | 91% |  |
| LDL-C Change value | - | - | - | - |
| ≤1mmol/L | 87.71 | [58.54, 116.87] | 91% | 0.521 |
| ＞1mmol/L | 102.97 | [66.63, 139.31] | 97% |  |
| **FCT Baseline ＞65μm** | - | - | - | - |
| LDL-C Follow-up value | - | - | - | - |
| ≤55 | 35.03 | [-0.19, 70.26] | 94% | 0.657 |
| 55-70 | 57.40 | [8.37, 106.43] | 93% |  |
| 70-100 | 39.59 | [23.32, 55.85] | 92% |  |
| 100-130 | 19.58 | [-17.56, 56.71] | 88% |  |
| LDL-C Change value | - | - | - | - |
| ≤1mmol/L | 24.78 | [9.64, 39.93] | 90% | 0.088 |
| ＞1mmol/L | 45.77 | [27.04, 64.51] | 94% |  |
